# Admixture and breed traceability in European indigenous pig breeds and wild boar using genome-wide SNP data

**DOI:** 10.1038/s41598-022-10698-8

**Published:** 2022-05-05

**Authors:** Christos Dadousis, Maria Muñoz, Cristina Óvilo, Maria Chiara Fabbri, José Pedro Araújo, Samuele Bovo, Marjeta Čandek Potokar, Rui Charneca, Alessandro Crovetti, Maurizio Gallo, Juan María García-Casco, Danijel Karolyi, Goran Kušec, José Manuel Martins, Marie-José Mercat, Carolina Pugliese, Raquel Quintanilla, Čedomir Radović, Violeta Razmaite, Anisa Ribani, Juliet Riquet, Radomir Savić, Giuseppina Schiavo, Martin Škrlep, Silvia Tinarelli, Graziano Usai, Christoph Zimmer, Luca Fontanesi, Riccardo Bozzi

**Affiliations:** 1 Dipartimento Di Scienze e Tecnologie Agrarie, Alimentari, Ambientali e Forestali, Università Di Firenze, 50144 Firenze, Italy; 2 Departamento Mejora Genética Animal, Instituto Nacional de Investigación y Tecnología Agraria y Alimentaria (INIA-CSIC), Crta. de la Coruña, km. 7, 5, 28040, Madrid, Spain; 3 Centro de Investigação de Montanha (CIMO), Instituto Politécnico de Viana Do Castelo, Escola Superior Agrária, Refóios do Lima, 4990‑706 Ponte de Lima, Portugal; 4 Department of Agricultural and Food Sciences, Division of Animal Sciences, University of Bologna, Viale Fanin 46, 40127 Bologna, Italy; 5 Kmetijski inštitut Slovenije, Hacquetova 17, 1000 Ljubljana, Slovenia; 6 MED-Mediterranean Institute for Agriculture, Environment and Development & Escola de Ciências E Tecnologia, Universidade de Évora, Pólo da Mitra, Ap. 94, 7006-554 Évora, Portugal; 7 Associazione Nazionale Allevatori Suini (ANAS), Via Nizza 53, 00198 Rome, Italy; 8 Department of Animal Science, Faculty of Agriculture, University of Zagreb, Svetošimunska c. 25, 10000 Zagreb, Croatia; 9 Faculty of Agrobiotechnical Sciences Osijek, Josip Juraj Strossmayer University of Osijek, Vladimira Preloga 1, 31000 Osijek, Croatia; 10 IFIP Institut du Porc, La Motte au Vicomte, BP 35104, 35651 Le Rheu Cedex, France; 11 Programa de Genética y Mejora Animal, Institute for Research and Technology in Food and Agriculture (IRTA), Torre Marimon, 08140 Caldes de Montbui, Barcelona, Spain; 12 Department of Pig Breeding and Genetics, Institute for Animal Husbandry, 11080 Belgrade‑Zemun, Serbia; 13 Animal Science Institute, Lithuanian University of Health Sciences, Baisogala, Lithuania; 14 Génétique Physiologie Et Systèmes d’Elevage (GenPhySE), Université de Toulouse, INRA, Chemin de Borde‑Rouge 24, Auzeville Tolosane, 31326 Castanet Tolosan, France; 15 Faculty of Agriculture, University of Belgrade, Nemanjina 6, 11080 Belgrade‐Zemun, Serbia; 16 AGRIS SARDEGNA, Loc. Bonassai, 07100 Sassari, Italy; 17 Bäuerliche Erzeugergemeinschaft Schwäbisch Hall, Schwäbisch Hall, Germany

**Keywords:** Comparative genomics, Conservation genomics, Agricultural genetics, Animal breeding, Genomics, Genetics, Genetic markers

## Abstract

Preserving diversity of indigenous pig (*Sus scrofa*) breeds is a key factor to (i) sustain the pork chain (both at local and global scales) including the production of high-quality branded products, (ii) enrich the animal biobanking and (iii) progress conservation policies. Single nucleotide polymorphism (SNP) chips offer the opportunity for whole-genome comparisons among individuals and breeds. Animals from twenty European local pigs breeds, reared in nine countries (Croatia: Black Slavonian, Turopolje; France: Basque, Gascon; Germany: Schwabisch-Hällisches Schwein; Italy: Apulo Calabrese, Casertana, Cinta Senese, Mora Romagnola, Nero Siciliano, Sarda; Lithuania: Indigenous Wattle, White Old Type; Portugal: Alentejana, Bísara; Serbia: Moravka, Swallow-Bellied Mangalitsa; Slovenia: Krškopolje pig; Spain: Iberian, Majorcan Black), and three commercial breeds (Duroc, Landrace and Large White) were sampled and genotyped with the GeneSeek Genomic Profiler (GGP) 70 K HD porcine genotyping chip. A dataset of 51 Wild Boars from nine countries was also added, summing up to 1186 pigs (~ 49 pigs/breed). The aim was to: (i) investigate individual admixture ancestries and (ii) assess breed traceability via discriminant analysis on principal components (DAPC). Albeit the mosaic of shared ancestries found for Nero Siciliano, Sarda and Moravka, admixture analysis indicated independent evolvement for the rest of the breeds. High prediction accuracy of DAPC mark SNP data as a reliable solution for the traceability of breed-specific pig products.

## Introduction

The process of domestication of pigs and the spread of the species around the world has been the subject of some studies in the recent past^1,2^, demonstrating that pig domestication involved multiple pig populations including wild boars^3,4^. The domestication aspects have often been investigated by the study of mitochondrial DNA, while genetic diversity was initially studied using simple sequence repeat (SSR) and amplified fragment length polymorphism (AFLP) in intensively selected breeds^5,6^ but also in indigenous populations of limited diffusion^7–10^. The development of single nucleotide polymorphisms (SNPs) panels with SNPs distributed across the entire genome provided new opportunities to investigate and decipher the complex relationship between indigenous pig breeds^11,12^. This is a topic that has to be taken on in order to enhance the safeguard of local pig populations. Considering the region of Europe and Caucasus, the Food and Agriculture Organization (FAO) identified 48 already extinct pig breeds, representing ~ 20% of the global pig breeds. Among the existing breeds in the region, 14 breeds are classified at critical risk of extinction, 5 are in a critical-maintained status, 24 are endangered, 11 are defined as endangered-maintained and 6 in a vulnerable situation (http://www.fao.org/dad-is/risk-status-of-animal-genetic-resources/en/). This means that more than 25% of the local European pig population is in a worrisome demographic status. The improvement of breeding and conservation programs for these indigenous breeds is becoming extremely important for multiple reasons. Firstly, it is well known that indigenous breeds are well-adapted to their local environment and are a unique genetic pool that might be essential, not only as pig biobank^13^, but also for the sustainability of the global pork chain. In addition, local pig farming is strongly related to niche products of high quality, which contribute to the local economy development and sustainability^14^. No less important is the increasing demand for organic and high welfare animal-based food products^15^, which has led consumers to prefer local breed products that are considered more nutritious, tasty, healthy and safe^16^ and because animals are usually reared freely and outdoors^17^.

It is important to note that the European pork production amounts on 21—22 thousand tonnes of meat per year (https://ec.europa.eu/eurostat/databrowser/view/tag00042/), heavily based on the use of cosmopolitan pig breeds. Moreover, Germany, Spain, France, Poland, and the Netherlands are the largest consumers in Europe. In this context, a powerful system to ensure pig breed traceability is required, that will enable products from pure local breeds to be clearly differentiated from their cosmopolitan counterparts and controlling fraud. Currently, the administrative traceability is not infallible, and the possibility of errors and frauds exists. The use of genetic markers could overcome these limits^18^. Microsatellites and SNP have been mainly used for traceability purposes, with the latter nowadays prevailing over the former, presenting many advantages such as easier laboratory handling, low mutation rate, and better suitability for standardization^19^. Several SNP based studies, often using a runs of homozygosity approach, aimed to detect candidate genes which allowed the identification of a specific breed^20^ and/or focused on genomic regions which discriminated populations from each other^21^. A pairwise fixation index (F_ST_) distances method was used to differentiate indigenous from commercial pig populations^11,22,23^, and to determine breeds belonging to different production systems^24^. Moreover, SNP detection from genome wide sequencing was used to develop a SNP chip for discriminating between purebred or crossbred Iberian origin of live pigs, meat and dry-cured pig products^25^. Other methods applied to distinguish breeds from each other are the investigation of the proportion of ancestry shared among the breeds^26,27^ and the clustering of genetically related individuals by discriminant analysis^28^. This latter method has been applied to trace sheep, using sets of SNPs able to separate breeds belonging to different geographic areas^29^ and for assigning animals to their true population^30^. A similar approach has been applied in cattle, where Dimauro et al.^31^ argued that the canonical discriminant analysis was able to efficiently distinguish the three breeds studied (Holstein, Brown Swiss, and Simmental). Moreover, various other methods exist in human^32^ and animal studies^33–35^ to identify a small set of ancestry informative SNPs, derived from genotyping or sequence data, that are helpful for population identification and breed traceability.

To the best of our knowledge no similar studies have been performed in pig breeds. In this work, we describe a comprehensive approach of using principal component analysis (PCA), admixture and discriminant analysis of principal components (DAPC) to evaluate pig breeds (indigenous and commercial) and wild boars traceability via the whole set of SNPs revealed by the GGP Porcine HD Array. The last two methods allow to predict the breed of origin (DAPC), and the proportions of ancestry per pig (admixture analysis).

## Results

### Population stratification and ancestry

**Figure 1:**
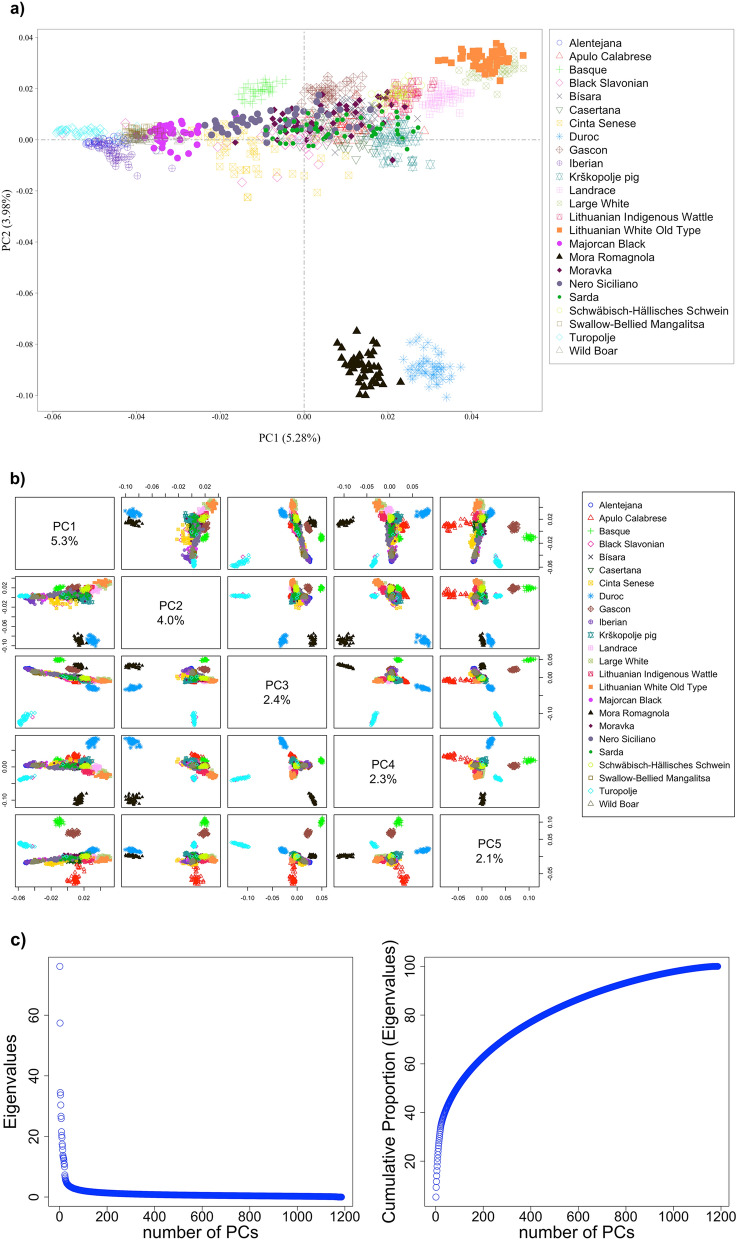
Results of the principal component analysis using the genotypes of 1,186 pigs: (**a**) Scatterplot of the first two principal components (PCs), (**b**) pairwise scatterplots of the first five PCs and (**c**) variance and cumulative variance explained by the PCs.

Analyses were based on 1,186 pigs and 40,364 SNP (Table 1). A PCA analysis was applied on the matrix of 1,186 pig genotypes. The scatterplots of the first two and all of the first five PCs in pairwise combinations are shown in Fig. 1a,b. Mora Romagnola and Duroc were clearly distinguished from the rest of the breeds (bottom-right quarter, Fig. 1a). Moreover, PC1 placed closely the Turopolje, Alentejana, Iberian*,* Swallow-Bellied Mangalitsa, Majorcan Black and Basque (left part, Fig. 1a). Lithuanian White Old Type and Large White were also separated in the opposite direction of PC1 (top-right quarter, Fig. 1a) and were closely positioned. In close proximity to those two was the Landrace breed. Considering PC1 and PC2, pigs belonging to the rest of the breeds were largely overlapped showing considerable within breed variation. Despite this, Gascon was almost clearly differentiated and this differentiation was more profound in PC5 (Fig. 1b). Considering further axes, Basque and Apulo Calabrese were also distinguished (PC3 and PC5, respectively), while Turopolje was further separated. It should be noted however, that the eigenvalues where low, with the first 2 eigenvalues accounting cumulatively ~ 9.3% of the original variability, while the first 697 eigenvalues captured ~ 90% (Fig. 1c).

**Table 1:** Breed name, type, country of origin and number of pigs analysed before (pre-) and after (post-) quality control (QC) per breed.

Breed name	Country of origin	N. pre-QC	N. post-QC
**Indigenous**			
Alentejana	Portugal	48	48
Apulo Calabrese	Italy	53	53
Basque	France	39	39
Bísara	Portugal	49	49
Black Slavonian (Crna Slavonska)	Croatia	49	49
Casertana	Italy	55	53
Cinta Senese	Italy	54	54
Gascon	France	48	48
Iberian	Spain	48	48
Krškopolje pig	Slovenia	52	52
Lithuanian Indigenous Wattle	Lithuania	48	48
Lithuanian White Old Type	Lithuania	48	48
Majorcan Black	Spain	48	48
Mora Romagnola	Italy	48	48
Moravka	Serbia	50	50
Nero Siciliano	Italy	50	48
Sarda	Italy	49	48
Schwäbisch-Hällisches Schwein (Swabian Hall pig)	Germany	51	49
Swallow-Bellied Mangalitsa	Serbia	50	50
Turopolje	Croatia	50	50
**Commercial**			
Duroc	Italy, Spain	53	53
Landrace	Italy, Spain	52	52
Large White	Italy, Spain	52	50
**Wild**			
Wild Boar	Finland, Greece, Hungary, Italy, Spain, Poland, Russia, The Netherlands, Tunisia	160	51

**Figure 2:**
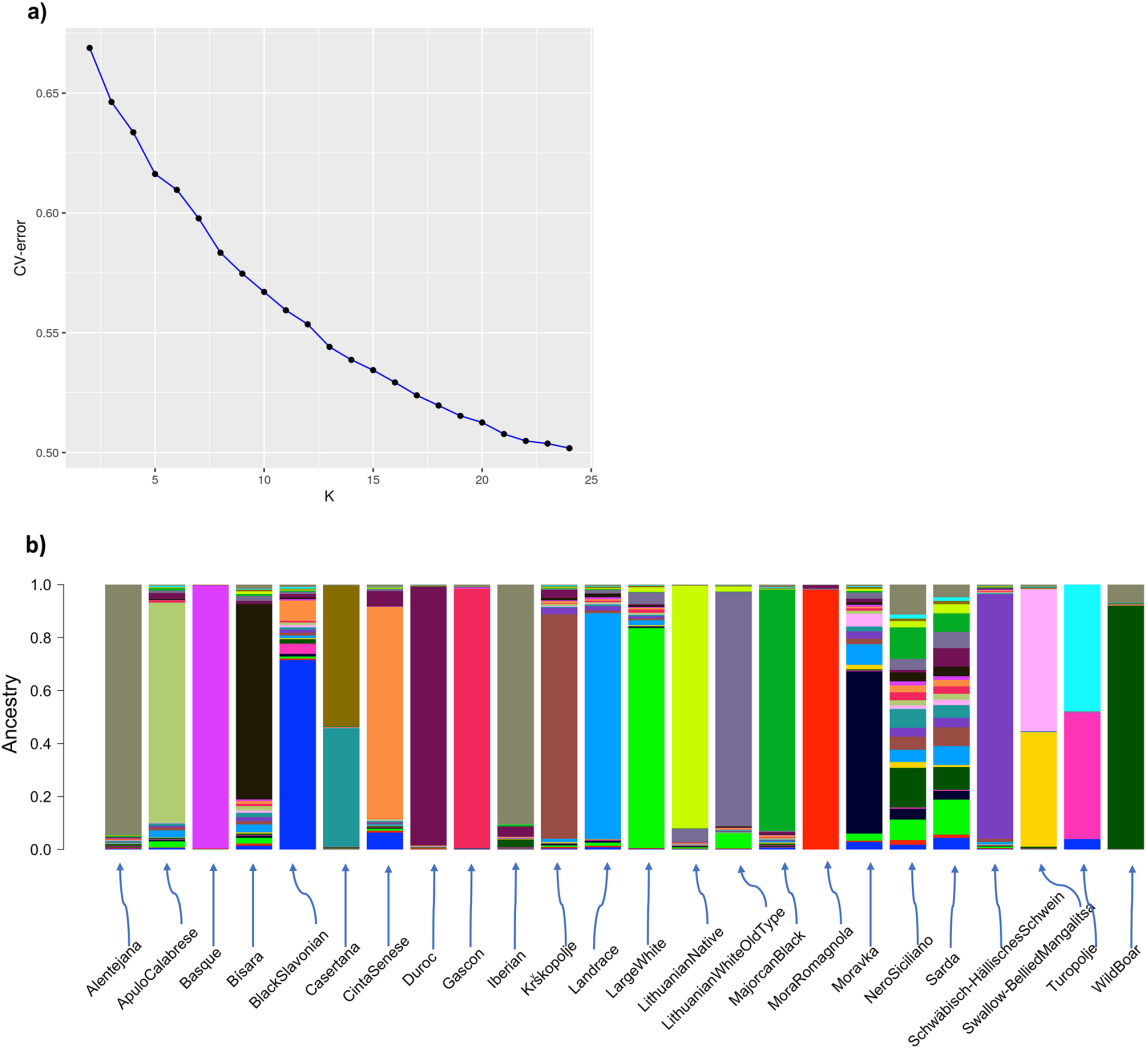
Results of admixture analysis: (**a**) fivefold cross-validation minimum error from K = 2–24; (**b**) summary per breed of admixture ancestries at K = 24.

Complementary to PCA, an admixture analysis was carried out, to estimate the proportion of ancestries per pig (Fig. S1). After cross-validation (CV), the model with 24 distinct groups was kept for further analysis (Fig. 2a). Results could be summarized in four main points (Fig. 2b, Fig. S1, S2 and Table S2): (i) in general, Alentejana, Basque, Gascon, Iberian and Mora Romagnola as indigenous breeds, and Duroc as commercial breed, showed the lowest levels of introgression, (ii) Casertana, Swallow-Bellied Mangalitsa and Turopolje consisted mainly of two group ancestries, (iii) the Italian breeds Nero Siciliano and Sarda showed a mosaic of different ancestries and (iv) Wild Boar ancestry contribution was mainly found in the Alentejana, Black Slavonian, Iberian, Nero Siciliano, Sarda and Swallow-Bellied Mangalitsa breeds.

### Discriminant analysis

#### Scenario 1 (semi-supervised learning)

**Figure 3:**
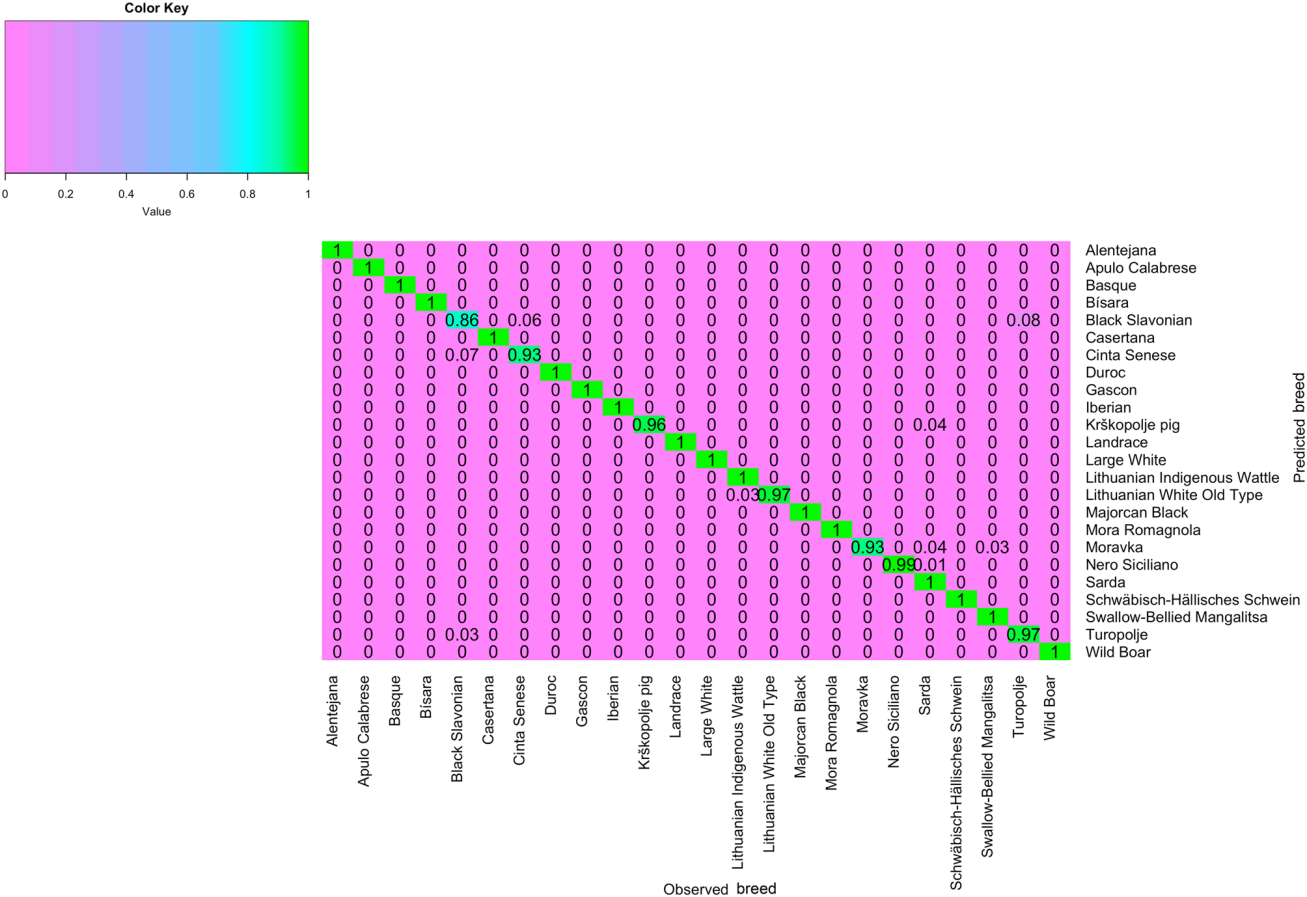
Heatmap of the DAPC assignment in the semi-supervised scenario with percentage of correct assignment per breed (in a scale of 0–1). Heatmap was constructed using the R^36^ package gplots^37^ and the function *heatmap.2*.

The overall successful assignment of pigs in breed of origin of the DAPC, averaged over the ten replicates, was 0.98 [0.967, 0.996] (Table 2). The number of PCs kept for DAPC ranged from 100 to 250 (52.4 and 69.4% of the original variance captured from the PCs, respectively). However, the number of PCs selected only marginally influenced the assignment success. The assignment success varied among breeds, with Black Slavonian, Cinta Senese, Krškopolje pig, Lithuanian White Old Type, Moravka, Nero Siciliano and Turopolje having < 100%, and the remaining breeds showing 100% accuracy (Fig. 3). The lowest value was observed for Black Slavonian (86%) with some pigs assigned to either as Cinta Senese or Turopolje (6 and 8%, respectively).

**Table 2:** Summary results of the DAPC model on the complete dataset.

Replicate	Assignment success, %	nPCs	VarPCs, %
rep1	0.983	100	0.524
rep2	0.988	200	0.646
rep3	0.975	100	0.525
rep4	0.979	200	0.649
rep5	0.992	100	0.526
rep6	0.988	100	0.526
rep7	0.996	250	0.695
rep8	0.967	200	0.649
rep9	0.992	200	0.646
rep10	0.975	250	0.694

nPCs = number of principal components selected for the DAPC model; VarPCs = percentage of original variance explained by the selected principal components. The total number of pigs was 1,186, the number of pigs in the TRN set was 944, and the number of pigs in the validation set was 242.

**Figure 4:**
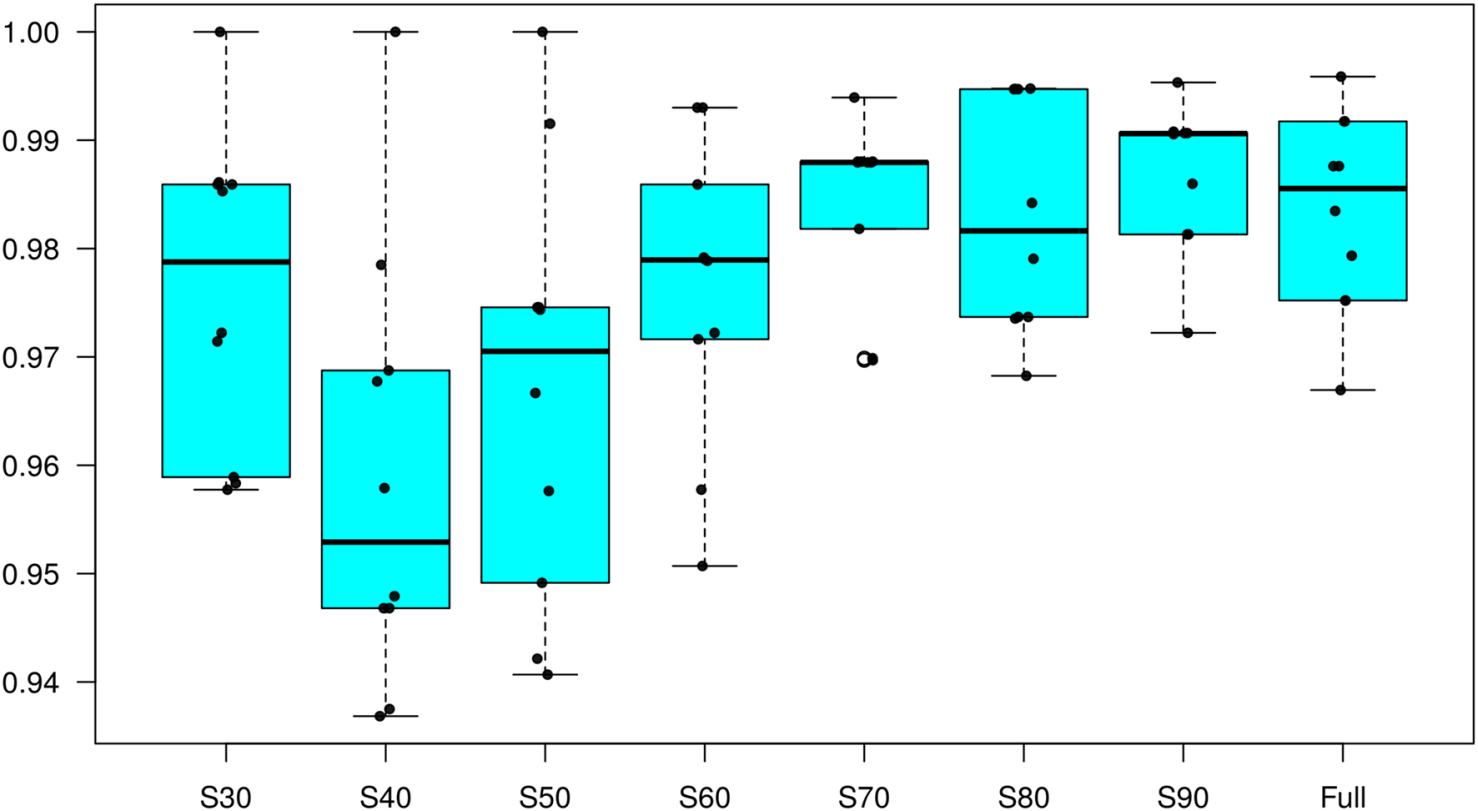
Boxplot of the overall successful assignment over different sampling (S) proportions of the data (30 to 100%) using DAPC. Median (black horizontal lines within the boxplots) over ten replicates (black dots).

In general, a positive effect of the sample size on the correct assignment of the DAPC model was found (Fig. 4). Although the mean model accuracy was slightly influenced by sample size, implying the robustness of the DAPC analysis, increasing sample size produced higher mean accuracies and reduced variance.

#### Scenario 2 (un-supervised learning)

**Figure 5:**
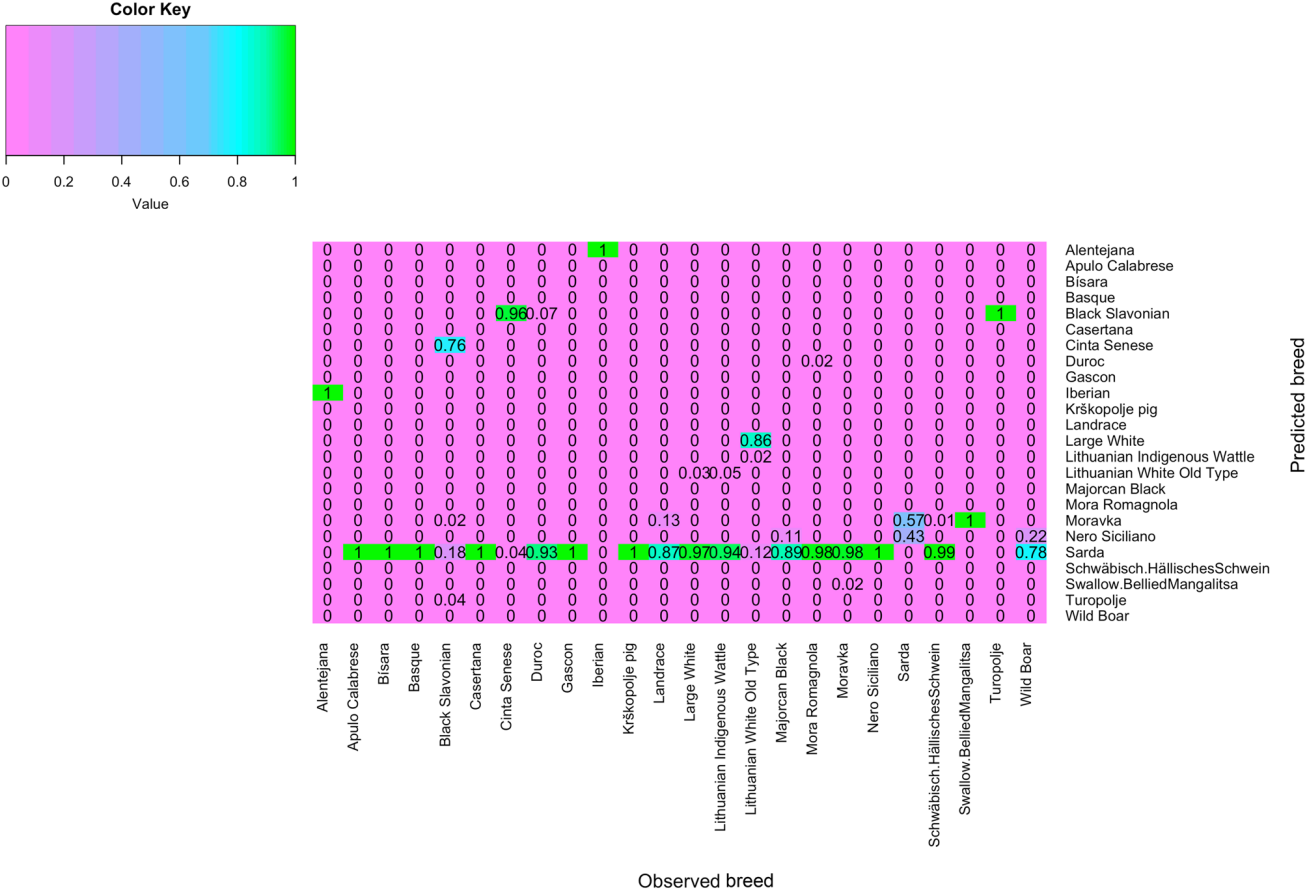
Heatmap of the DAPC assignment in the un-supervised scenario with percentage of external assignment per breed (in a scale of 0 to 1). Heatmap was constructed using the R^36^ package gplots^37^ and the function *heatmap.2*.

In the second scenario, VAL sets consisted of separate breeds and the evaluated breed was entirely excluded from the TRN set, hence the pigs were assigned to the rest of the 23 breeds. Results (Fig. 5) could be summarized in the following points: (i) some breeds were 100% assigned to only one breed (Alentejana, Apulo Calabrese, Basque, Bísara, Casertana, Gascon, Iberian, Krškopolje pig, Nero Siciliano and Turopolje), (ii) Cinta Senese, Duroc, Landrace, Large White, Majorcan Black, Mora Romagnola, Moravka, Sarda, Schwabisch-Hällisches Schwein, Swallow-Bellied Mangalitsa and Wild Boar were assigned to two breeds, (iii) Black Slavonian, Lithuanian Indigenous Wattle and Lithuanian White Old Type were assigned to three breeds, (iv) when the evaluated set of pigs was assigned to more than one breed, Sarda always appeared as one of the assigned breeds, so presenting mostly the highest assignment rate (except in the case of Black Slavonian, Lithuanian White Old Type and Swallow-Bellied Mangalitsa), (v) Alentejana was 100% assigned to Iberian and the other way around. That was the only case found of such a relationship between two breeds. For instance, Apulo-Calabrese, Basque, Bísara, Casertana, Krškopolje, and Nero Siciliano matched 100% to Sarda, but Sarda pigs were aligned only to Moravka and Nero Siciliano, (vi) Wild Boar was assigned mainly to Sarda and a small number to Nero Siciliano. The second most frequent breed to be assigned was Moravka with Black Slavonian, Landrace, Sarda, Schwabisch-Hällisches Schwein and Swallow-Bellied Mangalitsa being assigned to this breed.

**Figure 6:**
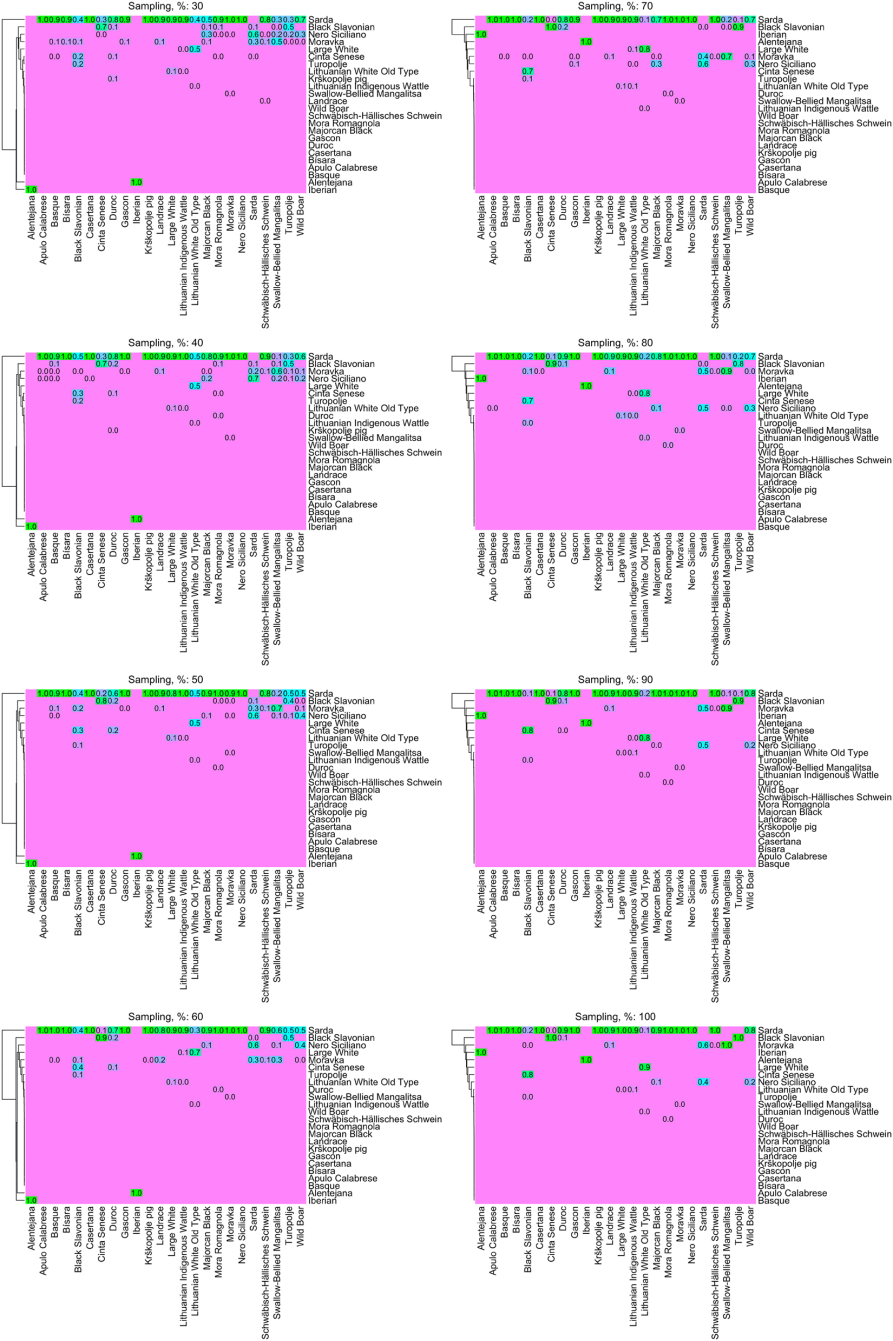
Heatmaps of the DAPC assignment in the un-supervised scenario, in increasing sample size, of percentage of external assignment per breed (in a scale of 0 to 1); x-axes show the observed and y-axes the predicted breed. Heatmaps were constructed using the R^36^ package ComplexHeatmap^38^.

These results were, in general, consistent and the sample size in the TRN set only marginally influenced the assignment of the breeds (Fig. 6). It is interesting that even with 30% of the dataset (~ 340 pigs), assignments were fairly consistent with results obtained utilizing the full dataset (~ 1,138 pigs). Sarda was in all subsets the breed mostly assigned. The percentage of classification of a specific breed to Sarda was either increased or decreased with an increasing sample size. For example, the proportion of the Black Slavonian classified as Sarda was medium (~ 40–50%) at a small sample size (30–60% of the data) and reduced to 10–20% with accumulated data, with the majority of the Black Slavonian pigs being assigned to Cinta Senese (~ 70–80%). Similarly, Lithuanian White Old Type had a ~ 40% assignment to Sarda and ~ 50% to Large White with ~ 340 pigs in the TRN set, and this ratio changed to 10–90% (Sarda and Large White, respectively) when all pigs from the remaining 23 breeds were considered in the TRN. In contrast, the percentage of Wild Boars assigned to Sarda was increased from 50 to 80% when increasing the sample size. The relationship between Alentejana – Iberian was not influenced in any scenario, resulting in 100% assignment of pigs of one breed to the other in all the cases.

## Discussion

Nowadays, modern pig farming worldwide is mostly highly intensive, utilizing few commercial breeds undergoing intense selection. Nevertheless, successful applications of indigenous pig farming exist, perhaps with the most prominent example being the Iberian pig in Spain. Disease outbreaks, such as the African swine-fever, threaten global pig production. Indigenous pig breeds consist of a unique genetic pool that might be proved of a great importance in the future, not only for the sustainability of the global pork chain but also for human research as in the case of the pig biobank^13,39^. However, indigenous pig farming is greatly based on outdoor rearing, making it vulnerable not only to disease outbreaks but also to natural disasters.

Studying genetic diversity is essential for the characterization of indigenous animal populations and can be used for conservation policies and promotion of local breeds. To support local pig farming, the TREASURE project joined researchers from nine countries and twenty-four research institutes to collect data from twenty European indigenous breeds. Previous genomic analyses of the aforementioned breeds were focused on linkage disequilibrium analysis and selection signatures detection using genome-wide SNP markers^12^, as well as genome sequencing data^40^. Studies on genetic diversity have also been performed, whether based on a candidate genes approach^41^ or a runs of homozygosity method^42^. The present work complements these studies by further investigating the proportion of ancestry shared among these breeds, together with three of the most representative commercial breeds as well as a joined dataset of Wild Boar, originating from nine countries. To address the question of potential breed traceability via genomic data, we further investigated the ability to predict the breed of origin by SNP markers. Linear discriminant analysis is a widely used methodology, but it lacks efficiency with high dimensional data such as genomic data. To overcome this problem, the methodology of linear discriminant analysis on a reduced dimensionality space, consisting of few principal components derived from SNP, was used.

PCA and admixture results were generally in agreement with high within-breed variability observed for the Sarda, Nero Siciliano and the Moravka, while Duroc and Mora Romagnola were the breeds that diverged most from the rest. Furthermore, unique ancestries were detected with both approaches for the Alentejana, Iberian, Basque, Duroc, Gascon and Mora Romagnola. Regarding Mora Romagnola, PCA and DAPC analyses showed contradictory results compared to previous study using candidate genes approach^41^. To explain this, it can be hypothesized that in a population such as Mora Romagnola, characterized by a low number of individuals and high level of inbreeding, there may be different response when investigating loci under selective pressure compared to neutral loci.

Nevertheless, slight differences among the PCA and admixture were also observed. For instance, the PCA scatterplot of the first two axes (Fig. 1a) clustered Turopolje close to Alentejana and Iberian; however, admixture analysis showed that ancestries were shared with Black Slavonian, Cinta Senese and Sarda (Fig. 2b, Table S2).

Regarding the closeness of some local with the cosmopolitan breeds as revealed by PCA (i.e., Duroc with Mora Romagnola; Large White with Lithuanian White Old Type), the reason for this could be the sharing some parts of the genome linked to phenotypic characteristics and origin of Lithuanian White pigs; however, the amount of variability explained by the first PCs is largely limited with respect to the overall genetic variability possessed by populations in the entire dataset. Moreover, although in PCA based on the scatterplot of the first two PCs (Fig. 1a) Duroc and Mora Romagnola were closely placed, the two breeds had common ancestries close to zero (Fig. 2b, Table S2).

Admixture analysis revealed common ancestries shared between some indigenous and the commercial breeds. More precisely, Duroc shared ancestries mainly with Cinta Senese, Iberian and Sarda; Landrace with Bísara, Moravka, Nero Siciliano and Sarda; and Large White with Lithuanian White Old Type, Nero Siciliano, Sarda, and Lithuanian Indigenous Wattle. Regarding Wild Boars, our dataset consisted of a set of 51 samples from seven European countries, Tunisia, and Russia, to capture as much variability and to avoid country-specific bias. Indeed, a recent study investigating the history of the domesticated European pigs indicated an interbreeding between the local pig breeds and Wild Boars^43^. Previous analysis on the same local breeds reported a close relationship, based on neighbour-joining tree constructed with Nei’s distances, between the Wild Boar and Alentejana and Iberian breeds^12^. In our analysis, introgression of Wild Boar was also found, besides the two aforementioned breeds, for the Italian breeds Nero Siciliano and Sarda. Common features between the PCA, admixture and the un-supervised DAPC were also observed, as explained below.

The un-supervised DAPC method could represent a real lab scenario for testing the “blind” or external to TRN set samples. In the un-supervised DAPC, many of the breeds, except Alentejana, Iberian, Black Slavonian, Cinta Senese, Lithuanian Wild Old Type and Turopolje, were mainly assigned as Sarda. This is not surprising, given the high admixture level of the Sarda breed. Black Slavonian was assigned to Cinta Senese in 76% of the cases, while Cinta Senese was predicted as Black Slavonian with 96% rate. Similarly, in the admixture analysis ~ 7.5% of the Black Slavonian was shared with Cinta Senese, while Turopolje was classified as Black Slavonian (100%). Interestingly, in the admixture analysis, Turopolje was assigned to two major ancestral groups sharing common ancestries mainly with Black Slavonian (Table S2). Regarding Lithuanian White Old Type, ancestries were mainly shared with Sarda (~ 6%), Lithuanian Indigenous Wattle (~ 5%) and Large White (~ 4.5%), so it would be expected to be predicted as Sarda. Nevertheless, the breed was assigned to a large extent to Large White (86%) followed by Sarda (~ 12%).

A second objective was to study traceability of pigs based on genome-wide SNP data. To resemble a practical application, the efficiency of the DAPC method was evaluated using an external validation. Furthermore, to assess the effect of sample size, the analyses were repeated several times with subsets of the dataset ranging from 30 to 90%. Although the correct assignment of the breeds was > 90% in all subsets, the variation of the correct assignment decreased with increased sample size, indicating a more robust model (Fig. 4). This level of correct reassignment of pigs is higher than the one reported by Muñoz et al.^41^, where there were many breeds with percentages of correct reassignment < 80%. Moreover, the actual differences might be even higher, since in that analysis an external validation was not considered and the whole data were analysed simultaneously. The correct reassignment was further improved for the Moravka, Nero Siciliano and Sarda breeds that had the lowest values in the DAPC analysis by Muñoz et al.^41^. However, in that study only a limited number of 39 SNPs in candidate genes was used.

Using the complete dataset, the majority of the breeds were correctly assigned to its breed of origin, with the exceptions of Black Slavonian, Cinta Senese, Krškopolje, Lithuanian White Old Type, Moravka and Turopolje, with the lowest value (86%) being observed for Black Slavonian (Fig. 3). In the case of Black Slavonian, there were some cases where animals were classified either as Cinta Senese or Turopolje. This was consistent with the shared ancestries found among the breeds, even at a low degree (Table S2). The relation among these breeds was further highlighted with the un-supervised DAPC, in which Black Slavonian was assigned mainly as Cinta Senese, followed by Sarda and Turopolje.

It should be noted that discrepancies between our results and previous genomic analyses on the same set of breeds were to some extent expected. There are two main reasons for this: (i) we considered three cosmopolitan breeds and a more diverse Wild Boar panel compared to Muñoz et al.^12^ and (ii) a whole-genome analysis was conducted compared to the candidate gene approach and the 39 SNP of Muñoz et al.^41^.

## Conclusion

We report a whole genome SNP analysis on admixed ancestries and classification of 20 European indigenous pig breeds, together with three commercial breeds and Wild Boars. Our results confirm previous analysis on the genomic diversity of the local breeds. Classification results using the 70 K HD porcine SNP chip were reliable and robust, hence DAPC could be considered as a potential tool for local pig breed traceability in the future. Our results indicate that robustness of the model could further benefit with bigger sample sizes. Nevertheless, cost of genotyping might be a limiting factor for a wide scale application. To overcome this limitation, a search for the minimum set of SNPs, that could achieve similar results obtained with the medium density SNP chip, could be proposed. Indeed, it would be useful to genotype a high proportion of the individuals belonging to the breeds with the highest risk of extinction or in any case with a greater risk of introgression from other populations. The cost of the set of SNPs is therefore fundamental given that for many of the breeds considered in this study there is a limited budget for genotyping. Our results suggest that integration of statistical methodologies to investigate genomic variability within and between breeds should be considered. We hope our findings to contribute and enhance the indigenous pig farming.

## Methods

### Animals and genomic data

Our initial pig genomic data (n = 1,195) were obtained from three sources: (i) 20 European indigenous breeds (n = 987) reared in 9 countries (Croatia: Black Slavonian, Turopolje; France: Basque, Gascon; Germany: Schwabisch-Hällisches Schwein; Italy: Apulo Calabrese, Casertana, Cinta Senese, Mora Romagnola, Nero Siciliano, Sarda; Lithuania: Indigenous Wattle, White Old Type; Portugal: Alentejana, Bísara; Serbia: Moravka, Swallow-Bellied Mangalitsa; Slovenia: Krškopolje pig; Spain: Iberian, Majorcan Black), and retrieved from the European funded project TREASURE (https://treasure.kis.si/). Blood samples were collected from each institution by specialized professionals, following standard guidelines. No interventions with animals were applied that would require ethical protocols (according to Directive 2010/63/EU-2010) (more details on sampling method can be found in Muñoz et al.^12^), (ii) three commercial breeds including Duroc (n = 53), Landrace (n = 52) and Large White (n = 52) and (iii) a sample of Wild Boars (n = 51) from Finland, Hungary, Italy, Spain, Poland, Russia, The Netherlands, Tunisia, and Greece was carefully selected from the Dryad Digital Repository: DOI: 10.5061/dryad.30tk6 (10.5061/dryad.30tk6)^44^. Further details on the selection of the Wild Boars are provided in the Supplementary Information. In addition, a small Spanish Wild Boar sample (n = 7) was also added^12^. All pigs from the indigenous and the three commercial breeds were genotyped with the GeneSeek Genomic Profiler (GGP) 70 K HD porcine genotyping chip containing 68,516 SNPs. The Wild Boars were genotyped with the Illumina 60 K SNP data^45^. The merged data contained 42,464 autosomal SNP. Samples with more than 10% and SNPs with more than 5% of missing values were excluded. The final data consisted of 1,186 pigs and 40,364 SNP (Table 1).

### Population stratification and ancestry

Admixture and PCA were used to investigate the data structure in terms of distinct populations. The two approaches, are complementary to each other. More precisely, PCA produces orthogonal projections of the original data, variance driven (from the highest to the lowest), focusing on how different populations are structured (between and within). In contrast, an admixture analysis provides the proportions from each of the source populations in each sample, i.e., how the individual samples are related to the source populations (ancestries). The PCA was performed in R software^36^, using the *prcomp* function, while the proportion of mixed ancestry was assessed using the *ADMIXTURE 1.22* software^46,47^. The number of ancestries (K) to be retained in admixture (K = 2–24) was evaluated via a fivefold cross-validation (CV) and the model with minimum CV error was selected for further analysis. Results were also summarized per breed for an easier representation.

### Discriminant analysis

DAPC^48^ was applied to assess breed traceability, as implemented in the R package *adegenet*^36,49,50^. DAPC replaces the original SNP data with a small set of principal components (PCs) and then applies a linear discriminant analysis on the selected PCs. In this way, DAPC maximizes the differences among groups while overlooking at the variability within groups. The number of PCs to be used in the discriminant analysis is determined via CV and the targeting function can be either the lowest root mean squared error or the highest mean success. To select the best option both methods were evaluated: In brief, data were randomly sampled in sets starting from 30% and augmenting by 10% up to the complete dataset, one repetition each, having all the breeds represented (stratified sampling), and the overall model assignment accuracy was recorded (Table S1). For each set, a tenfold CV was applied, and repeated 30 times, to select the optimum number of PCs for the discriminant analysis. On average, minimum prediction error slightly outperformed the highest mean success, and this was the option kept in subsequent analysis. It should be noted that according to Jombart^49^ this is also the recommended option.

The objective of DAPC was to represent real case scenarios, i.e., to identify an external individual membership to a group (external validation). In such a case, the discriminant function is developed in a training set (TRN) and then applied on genotypes of an external validation set (VAL). The function *predict.dapc* was used for this analysis. Two different approaches were applied:

- Scenario 1 (semi-supervised learning). Data were randomly (without replacement) split at 80–20% for the TRN-VAL set, and the split was repeated 10 times. Random sampling was conditioned such that all the breeds were present in both TRN and VAL sets (stratified sampling).
- Scenario 2 (un-supervised learning). Each breed was analysed separately and consisted of the VAL set. In this scenario, no pigs of the VAL set were present in the TRN set, hence pigs had to be classified in one of the other 23 breeds. The TRN set consisted of pigs from the rest of the 23 remaining breeds, randomly selected (without replacement). This procedure was repeated 10 times. Scenario 2 can be seen as a method to assess similarity among breeds.

In both scenarios, the design of the DAPC analysis included: (i) tenfold CV for the selection of the optimum number of the PCs, (ii) the maximum number of PCs tested was set to 300 and (iii) minimum prediction error as the target function for model selection. Results were summarized over the 10 repetitions. Moreover, to assess the effect of the sample size and the robustness of the model, the complete dataset was split in sets of 10% increase (from 30 up to 100%). The terms (semi/un)-supervised should not be confused with the terminology in machine learning. These terms were used to distinguish between the two scenarios of DAPC, and although they are analogous to same terms used in the statistical field of machine learning they are not identical.

## Supplementary Information

Supplementary Information 1.

Supplementary Information 2.

## Data Availability

The authors confirm that the data supporting the findings of this study are available within the article and its supplementary materials. The raw genetic datasets generated during the current study are available from the corresponding author on reasonable request. The external Wild Boars sample can be found in Dryad Digital Repository: DOI: 10.5061/dryad.30tk6 (10.5061/dryad.30tk6).
