# Supplementary material for: Admixture and breed traceability in European indigenous pig breeds and wild boar using genome-wide SNP data
Source: Sci Rep. 2022 May 5;12:7346. doi: 10.1038/s41598-022-10698-8 (PMC9072372; doi:10.1038/s41598-022-10698-8)
Supplement: Supplementary file 1 — Supplementary Information 1. [file 41598_2022_10698_MOESM1_ESM.pdf]

Appendix

Description of the external Wild Boar data included in the analysis

Selection of the external Wild Boar (WB) pigs was done from the Dryad Digital Repository: DOI: doi:10.5061/dryad.30tk6 (http://dx.doi.org/10.5061/dryad.30tk6), including 2,113 individuals. From those, only pigs of European, Russian and Tunisian origin were considered for analysis (n=160; Appendix Table 1). After filtering, a sample of WBs (n=51) from Finland, Hungary, Italy, Spain, Poland, Russia, The Netherlands, Tunisia, and Greece was merged with a small Spanish WB population (n=7). Principal component analyses (PCA) were used to identify potential outliers (i.e., individuals that were grouped to another breed form what was officially registered) that could lead to false results in the subsequent admixture and discriminant analysis on principal components (DAPC) analysis.

Appendix Table 1. Description of the 160 wild boars.

Country Full Name	Breed Full Name	Breed Acronym	Description	Number_of_Individuals
Croatia	Croatia_WildBoar	HRWB	European_Wild	16
Finland	Finland_WildBoar	FIWB	European_Wild	5
France	NW_European_WildBoar	NEWB	European_Wild	6
Greece	Greece_Samos_WildBoar	GRWB	European_Wild	7
Greece	South_Balkan_WildBoar	SBWB	European_Wild	20
Italy	Italy_Sardinia_WildBoar	ITWB2	European_Wild	20
Italy	Italy_WildBoar	ITWB1	European_Wild	19
Netherlands	NW_European_WildBoar	NEWB	European_Wild	14
Poland	Poland_WildBoar	PLWB	European_Wild	8
Portugal	Iberian_WildBoar	IBWB	European_Wild	18
Russia	Russia_Wildboar_West	RUWB3	European_Wild	20
Tunisia	Tunisia_WildBoar	TNWB	African_Wild	7

The filtering procedure is described below:

1. A PCA was conducted on the 160 WB genotypes. By plotting PC1 vs. PC2, the Russian of Asian origin were clearly separated from the rest and 1 Greek from island was grouped with the Greek continental WBs (Appendix Figure 1a). Moreover, by plotting PC3 vs. PC5, 4 continental Italian and 2 Italian Sardinian WBs were separated from their group.

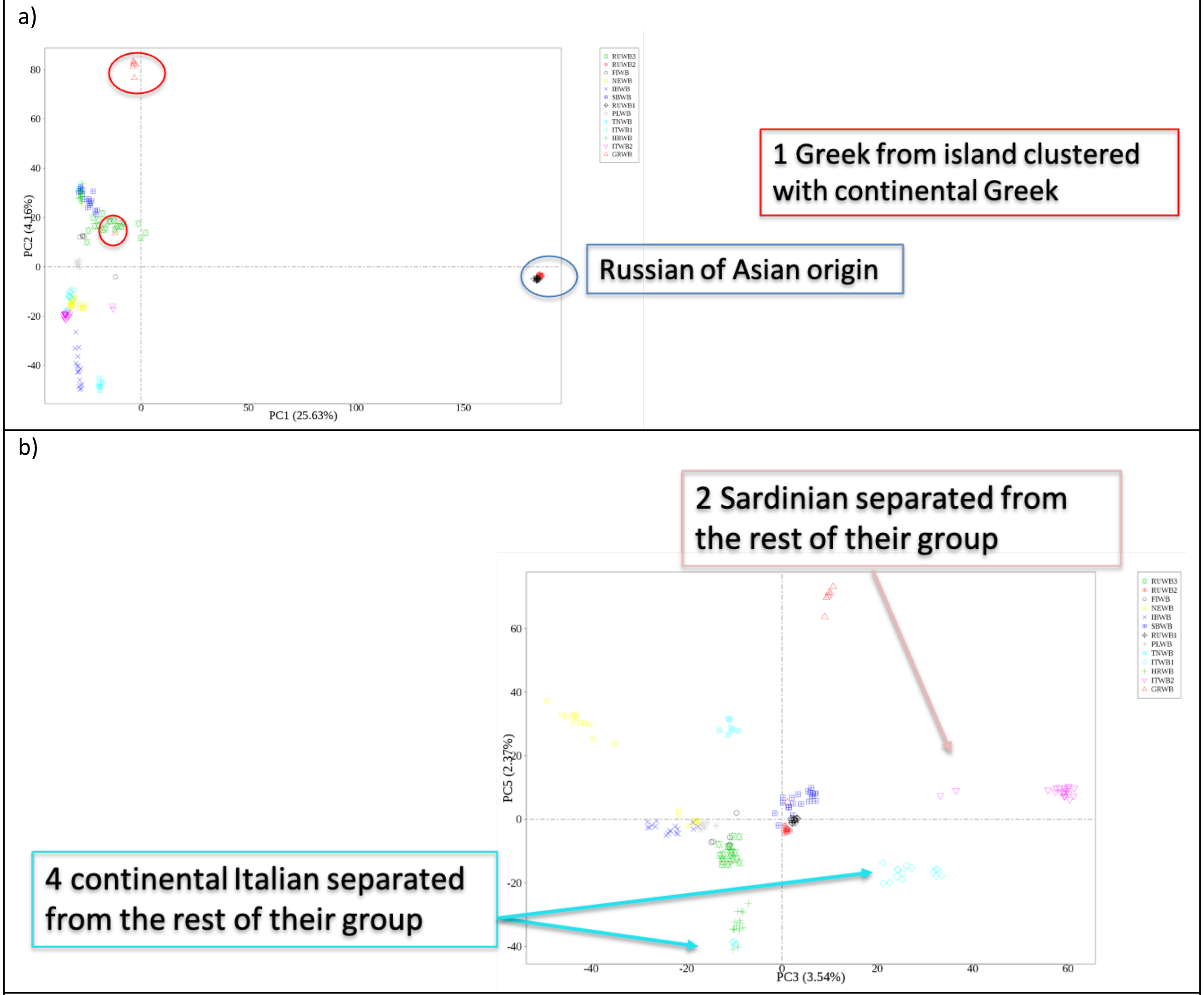

Appendix Figure 1. First principal component analysis (PCA) on the 160 wild boar genotypes; scatterplot of the a) first 2 principal components (PCs) and b) PC3 vs. PC5.

2. The Russian outliers were excluded and a PCA was conducted (Appendix Figure 2a,b). One Finish was grouped with Polish WB; 6 WBs of Dutch and French origin were separated from their breed of origin; 4 continental Italian and 2 Italian Sardinian WBs were separated from the rest of their group as in step1; 1 Greek from island was grouped with the Greek continental WBs as in step 1.

a)

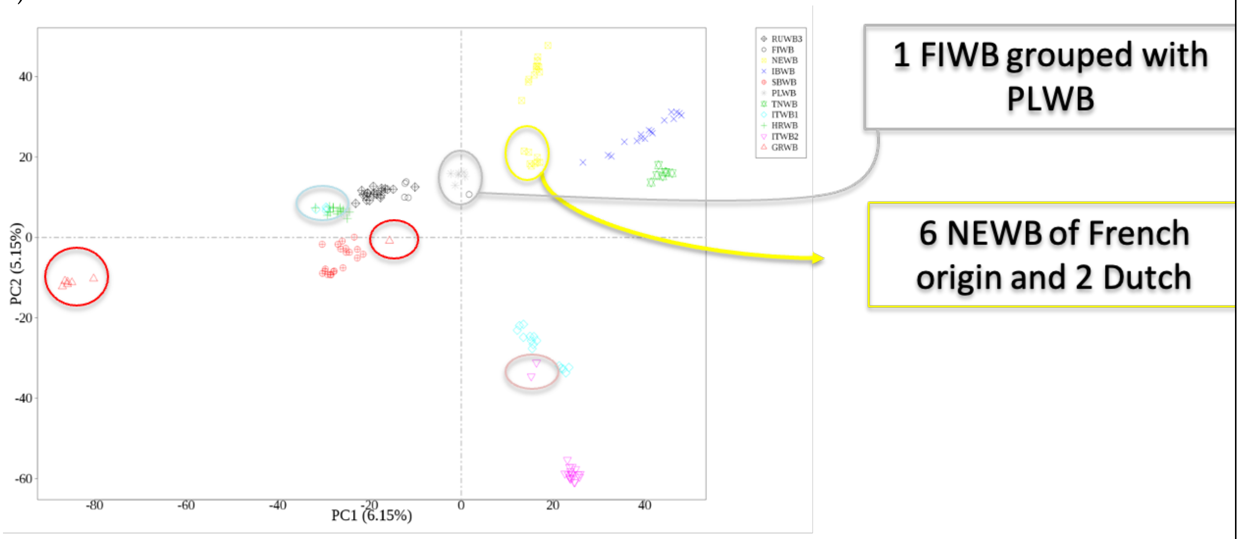

b)

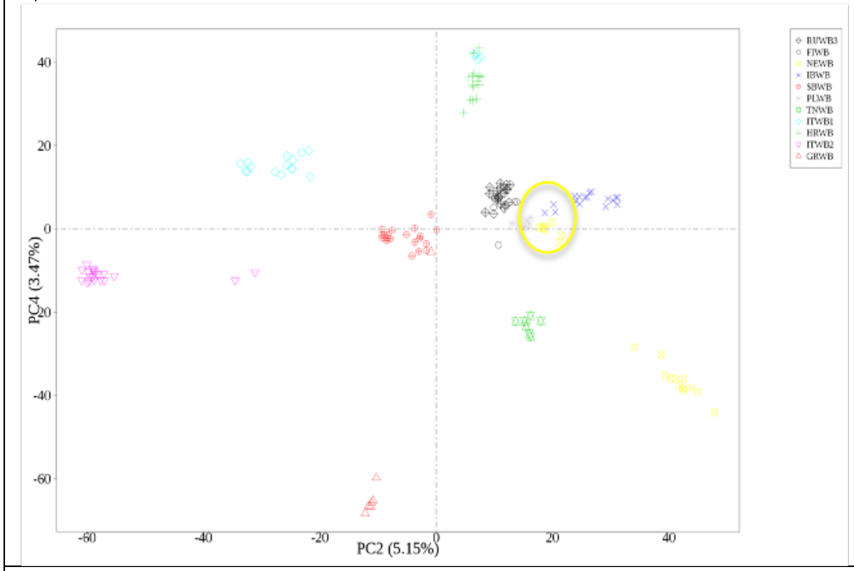

Appendix Figure 2. Second principal component analysis (PCA) after removing the Russian outliers identified in step1; scatterplot of the a) first 2 principal components (PCs) and b) PC2 vs. PC4.

3. All of the outliers detected in step 2 were excluded and a PCA was conducted (Appendix Figure 3). After this step, a total of 141 WBs remained (Appendix Table 2).

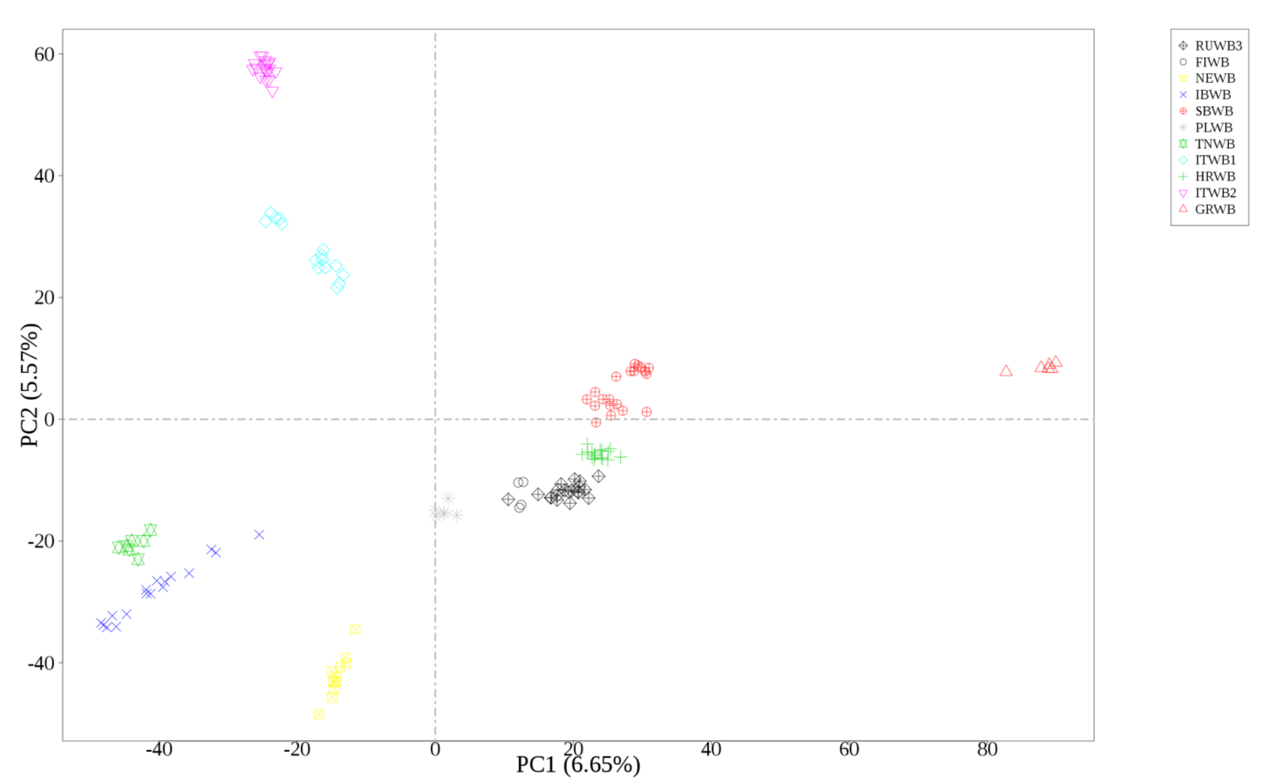

Appendix Figure 3. Third principal component analysis (PCA) after removing the outliers identified in step2; scatterplot of the a) first 2 principal components (PCs).

Country_FullName	Breed_FullName	PopName	Description	Number_of_Individuals
Croatia	Croatia_WildBoar	HRWB	European_Wild	16
Finland	Finland_WildBoar	FIWB	European_Wild	4
France	NW_European_WildBoar	NEWB	European_Wild	0
Greece	Greece_Samos_WildBoar	GRWB	European_Wild	6
Greece	South_Balkan_WildBoar	SBWB	European_Wild	20
Italy	Italy_Sardinia_WildBoar	ITWB2	European_Wild	18
Italy	Italy_WildBoar	ITWB1	European_Wild	15
Netherlands	NW_European_WildBoar	NEWB	European_Wild	12
Poland	Poland_WildBoar	PLWB	European_Wild	6
Portugal	Iberian_WildBoar	IBWB	European_Wild	17
Russia	Russia_Wildboar_West	RUWB3	European_Wild	20
Tunisia	Tunisia_WildBoar	TNWB	African_Wild	7

4. To have a balanced population size between the TREASURE pig breeds and the WBs, we randomly sampled 44 WBs conditionally on the country of origin (4 WBs each), such as all countries were represented. To the 44 external WBs, 7 Spanish WBs were also added, summing up to 51 WBs.

5. In total, 42,262 single nucleotide polymorphisms were in common between the TREASURE and the external WBs; with SNP positions of the WBs dataset being updated to the GeneSeek Genomic Profiler (GGP) 70 K HD, where needed.
