# Supplementary material for: Admixture and breed traceability in European indigenous pig breeds and wild boar using genome-wide SNP data
Source: Sci Rep. 2022 May 5;12:7346. doi: 10.1038/s41598-022-10698-8 (PMC9072372; doi:10.1038/s41598-022-10698-8)

Supplementary Materials

Supplementary Table S1. Model assessment based on minimum prediction error (LMSE) or the highest mean success (HMS).

Sample, %	N. Breed	N. IDs	N. PCs (HMS)	N. PCs (LMSE)	Var. PCs (HMS)	Var. PCs (LMSE)	Assign. Success (HMS)	Assign. Success (LMSE)
30	24	356	50	100	0.491	0.631	0.9775	0.9803
40	24	474	100	200	0.592	0.761	0.9852	0.9873
50	24	593	150	150	0.642	0.642	0.9882	0.9882
60	24	712	100	100	0.545	0.545	0.9831	0.9831
70	24	830	100	100	0.532	0.532	0.9855	0.9855
80	24	949	150	150	0.591	0.591	0.9884	0.9884
90	24	1067	100	200	0.518	0.634	0.9888	0.9897
100	24	1186	100	150	0.513	0.575	0.9890	0.9890
Average	/	/	/	/	/	/	0.9857	0.9865

Sample = percentage of data sampled at random; N. Breed = number of breeds analyzed in each sample; N. IDs = number of pigs analyzed in each sample; N. PCs = number of principal components (PCs) selected; Var. PCs = variance explained of the selected PCs; Assign. Success = overall model assignment success.

Supplementary Table S2 Averaged admixture ancestries per breed at K=24.

**Breed**	**V1**	**V2**	**V3**	**V4**	**V5**	**V6**	**V7**	**V8**	**V9**	**V10**	**V11**	**V12**	**V13**	**V14**	**V15**	**V16**	**V17**	**V18**	**V19**	**V20**	**V21**	**V22**	**V23**	**V24**
Alentejana	0.31	0.38	0.09	0.28	0.22	0.75	0.21	0.37	0.14	0.07	0.45	0.09	0.15	0.26	0.32	0.16	0.11	0.18	0.14	0.45	0.17	0.29	0.11	**94.31**
Apulo Calabrese	0.64	0.15	2.29	0.56	0.23	0.55	0.11	2.73	1.06	0.59	0.95	0.27	**83.13**	0.74	0.12	0.07	0.34	2.37	0.89	0.91	0.26	0.15	0.56	0.35
Basque	0.01	0.00	0.05	0.01	0.01	0.00	0.00	0.00	0.05	0.00	0.02	0.00	0.02	0.22	0.03	**99.39**	0.00	0.06	0.00	0.00	0.04	0.00	0.00	0.10
Bisara	1.47	0.78	2.15	0.80	0.25	0.59	0.41	**3.03**	1.21	1.57	1.61	0.93	1.55	0.84	1.10	0.64	**73.77**	1.19	1.84	0.75	0.98	0.65	0.29	1.61
Black Slavonian	**71.57**	0.56	0.77	0.98	**3.86**	1.65	0.40	0.99	1.09	1.14	0.81	0.93	0.96	0.61	**7.68**	0.57	0.48	1.39	0.83	0.46	0.43	0.30	0.59	0.94
Casertana	0.07	0.13	0.15	0.20	0.09	0.25	0.11	0.05	0.06	0.05	**44.64**	0.09	0.07	0.04	0.12	0.01	0.01	0.17	0.06	0.13	0.06	**53.11**	0.01	0.35
CintaSenese	**6.40**	0.65	0.82	0.39	0.09	0.52	0.15	0.52	0.33	0.33	0.58	0.12	0.59	0.07	**80.07**	0.16	0.10	**5.65**	0.83	0.12	0.35	0.17	0.17	0.84
Duroc	0.06	0.40	0.14	0.20	0.05	0.02	0.10	0.02	0.05	0.05	0.04	0.06	0.17	0.02	0.14	0.03	0.05	**97.70**	0.02	0.09	0.04	0.22	0.05	0.30
Gascon	0.08	0.05	0.04	0.05	0.04	0.03	0.02	0.02	0.00	0.09	0.09	0.04	0.02	**97.78**	0.10	0.57	0.02	0.05	0.12	0.11	0.02	0.03	0.02	0.60
Iberian	0.13	0.20	0.02	0.12	0.32	2.98	0.15	0.01	0.14	0.10	0.07	0.11	0.13	0.03	0.28	0.06	0.08	**3.73**	0.02	0.79	0.03	0.01	0.06	**90.43**
Krskopolje	0.40	0.53	0.58	0.56	0.14	0.16	0.39	1.35	**84.67**	2.55	0.32	0.31	0.76	0.19	0.88	0.34	0.87	2.99	0.42	0.27	0.32	0.36	0.35	0.32
Landrace	0.88	0.53	1.21	0.70	0.36	0.06	0.17	**85.33**	0.96	1.61	0.71	0.33	0.53	0.52	0.58	0.77	1.35	0.11	1.20	0.43	0.37	0.57	0.27	0.47
LargeWhite	0.27	0.30	**83.03**	0.53	0.15	0.08	0.38	1.86	0.55	0.94	0.56	0.11	0.68	1.19	0.60	0.33	0.74	0.38	**4.47**	0.11	1.79	0.52	0.22	0.25
Lithuanian Indigenous Wattle	0.13	0.18	0.52	0.47	0.13	0.09	0.18	0.07	0.06	0.04	0.07	0.12	0.16	0.27	0.10	0.07	0.13	0.08	**4.94**	0.17	**91.63**	0.08	0.03	0.29
Lithuanian White Old Type	0.12	0.28	**5.71**	0.07	0.07	0.00	0.09	0.32	0.26	0.19	0.21	0.07	0.16	0.17	0.42	0.12	0.40	0.13	**88.49**	0.10	1.94	0.24	0.06	0.39
MajorcanBlack	0.77	0.13	0.13	0.35	0.23	0.62	0.37	0.52	0.11	0.26	0.26	0.14	0.37	0.28	0.77	0.19	0.13	0.97	0.41	**91.13**	0.25	0.22	0.34	1.07
MoraRomagnola	0.02	**98.14**	0.01	0.00	0.04	0.00	0.06	0.13	0.00	0.00	0.02	0.00	0.03	0.00	0.00	0.00	0.02	1.34	0.00	0.01	0.02	0.02	0.00	0.14
Moravca	2.87	0.43	2.70	**61.22**	0.38	0.40	1.72	**7.74**	2.13	2.80	1.79	**4.80**	1.17	0.95	0.56	0.70	1.05	1.32	2.28	0.64	0.79	0.61	0.34	0.62
NeroSiciliano	1.79	1.74	**7.80**	**4.06**	0.46	**15.00**	2.23	**4.57**	**4.93**	**3.38**	**7.06**	1.38	1.92	**3.01**	2.63	1.54	**3.37**	0.93	**4.12**	**11.97**	2.36	0.93	1.54	**11.30**
Sarda	**4.40**	1.23	**13.18**	**3.32**	0.41	**8.63**	0.72	**7.18**	**7.05**	**3.58**	**4.82**	2.07	2.25	2.74	2.44	1.41	**3.57**	**7.04**	**6.16**	**6.99**	**3.46**	1.22	1.39	**4.75**
Schwabisch-Hallisches Schwein	0.25	0.26	0.65	0.27	0.15	0.06	0.18	1.05	1.21	**92.35**	0.38	0.13	0.24	0.33	0.37	0.36	0.29	0.03	0.30	0.23	0.22	0.26	0.10	0.36
Swallow-Bellied Mangalitsa	0.06	0.02	0.07	0.10	0.18	0.65	**43.31**	0.04	0.02	0.13	0.09	**53.58**	0.13	0.07	0.19	0.01	0.03	0.09	0.00	0.21	0.02	0.00	0.13	0.88
Turopolje	**3.88**	0.00	0.00	0.04	**47.77**	0.02	0.00	0.13	0.01	0.00	0.01	0.03	0.00	0.00	0.08	0.04	0.02	0.00	0.03	0.01	0.00	0.03	**47.86**	0.03
WildBoar	0.05	0.07	0.13	0.05	0.04	**91.75**	0.15	0.01	0.16	0.05	0.00	0.05	0.05	0.02	0.03	0.10	0.01	0.12	0.02	0.31	0.03	0.00	0.02	**6.79**

In bold values greater than 3%. Rows represent breed and columns the 24 group ancestries.

Supplementary Fig. S1. Individual pig ancestries at K=24.

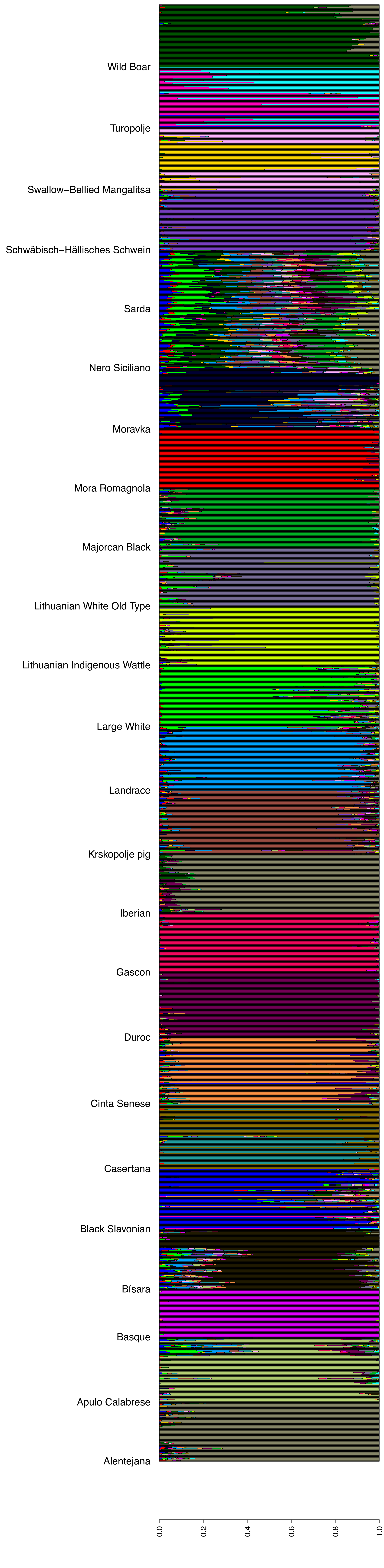

Supplementary Fig. S2. Change of average admixture ancestries per breed from K=2 to 24.

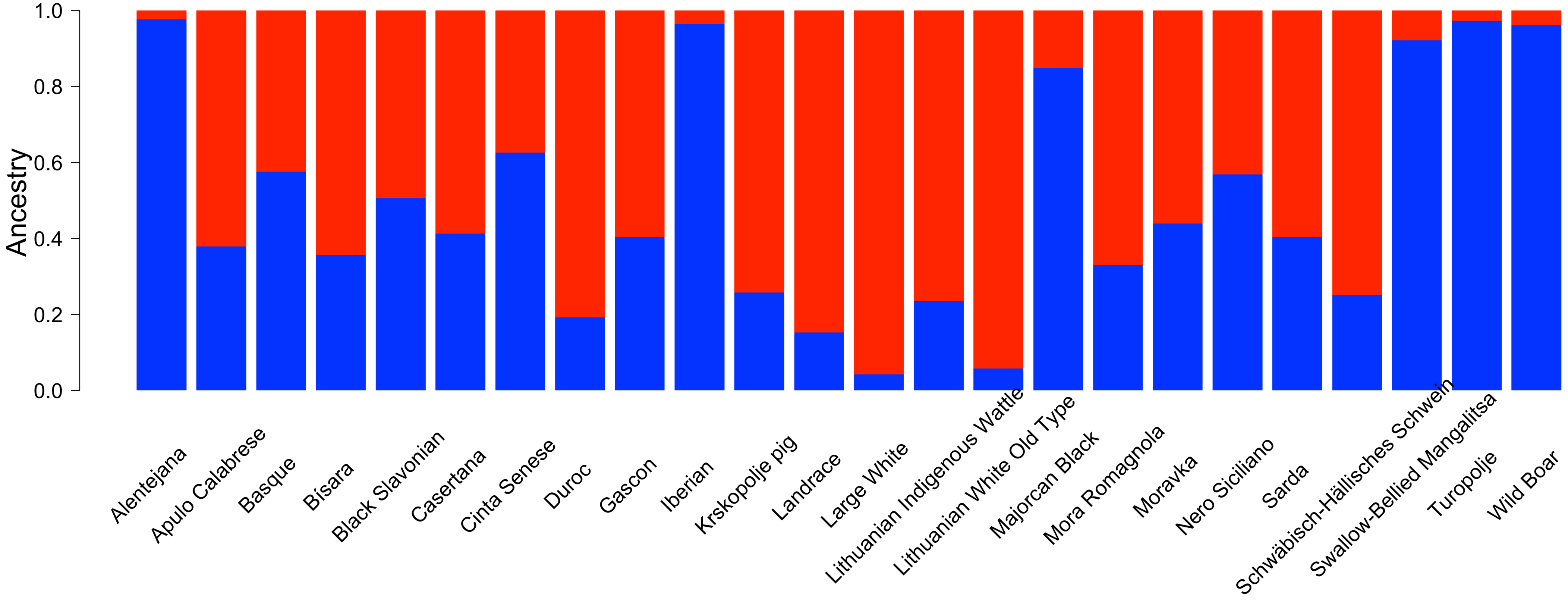

Supplementary Fig. S2. Change of average admixture ancestries per breed from K=3 to 24.

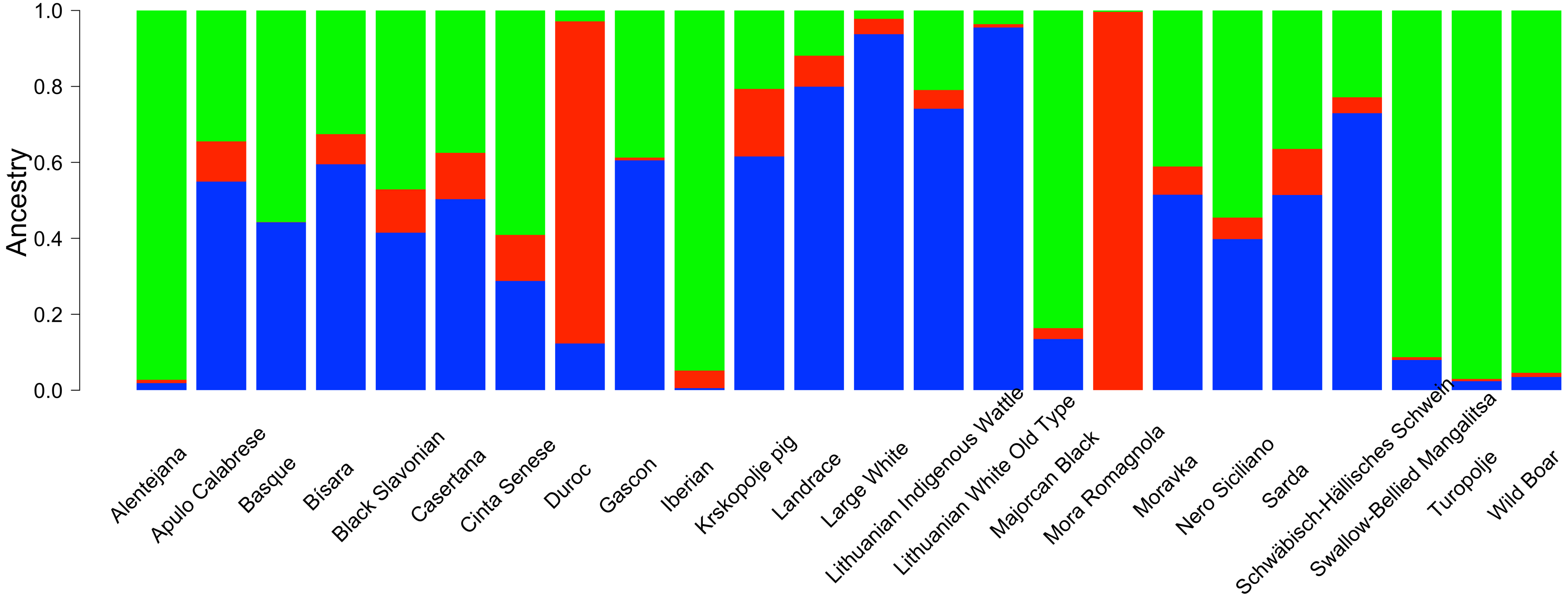

Supplementary Fig. S2. Change of average admixture ancestries per breed from K=4 to 24.

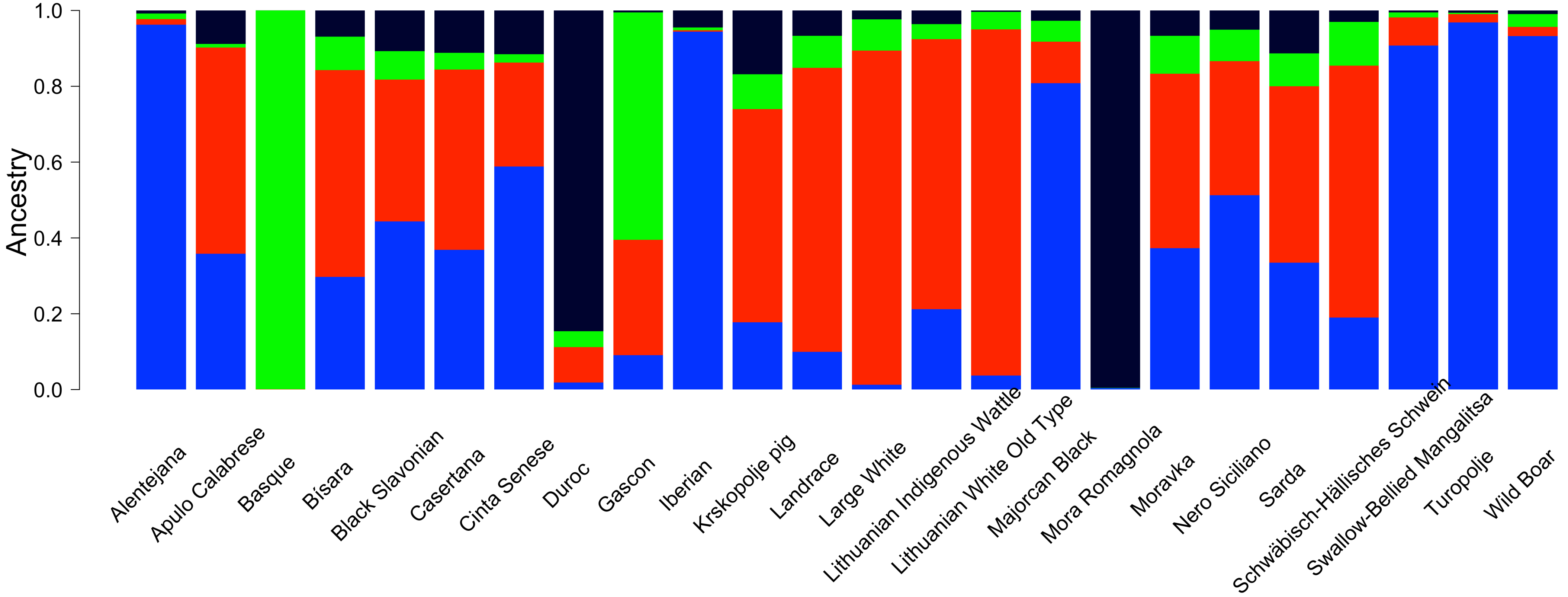

Supplementary Fig. S2. Change of average admixture ancestries per breed from K=5 to 24.

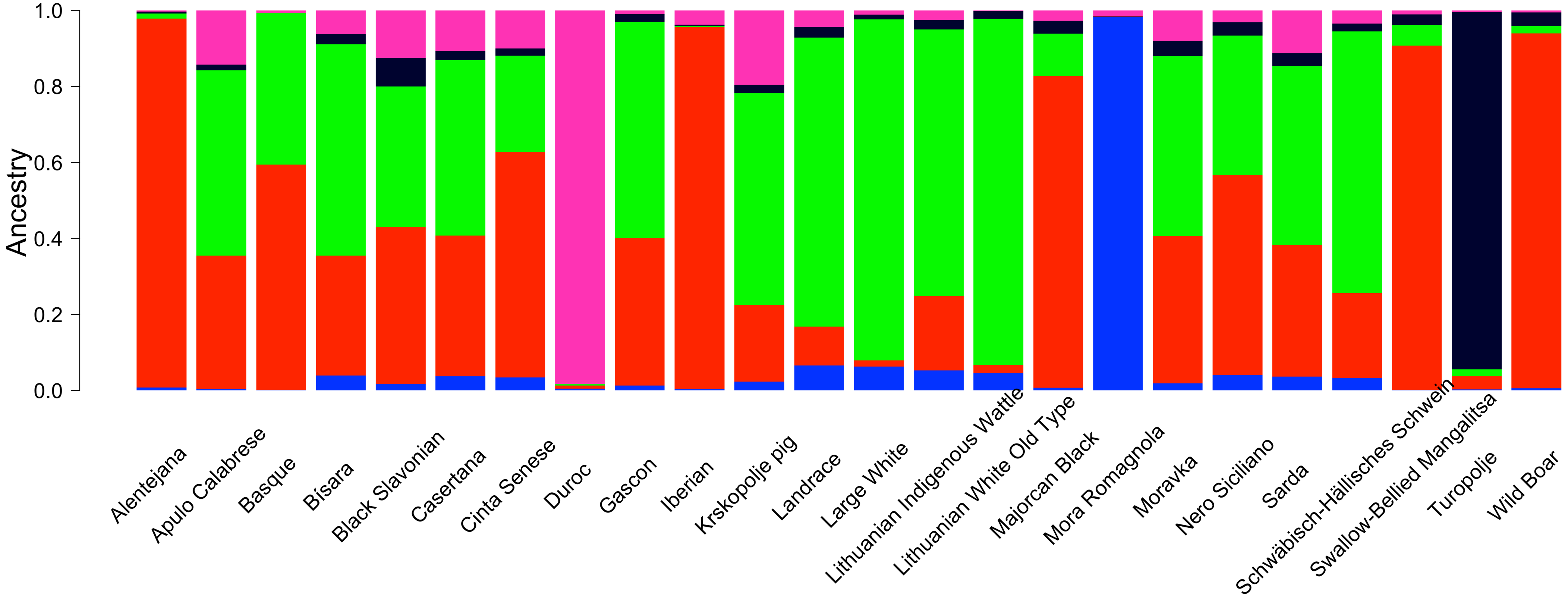

Supplementary Fig. S2. Change of average admixture ancestries per breed from K=6 to 24.

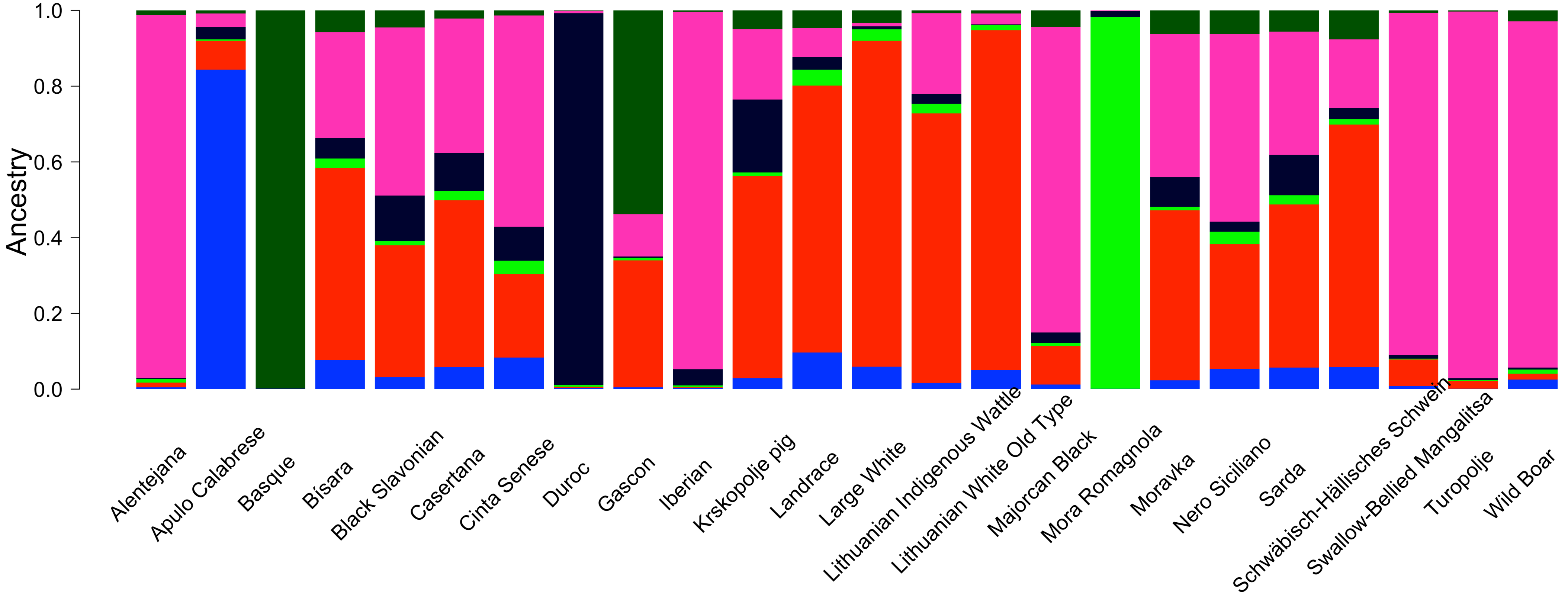

Supplementary Fig. S2. Change of average admixture ancestries per breed from K=7 to 24.

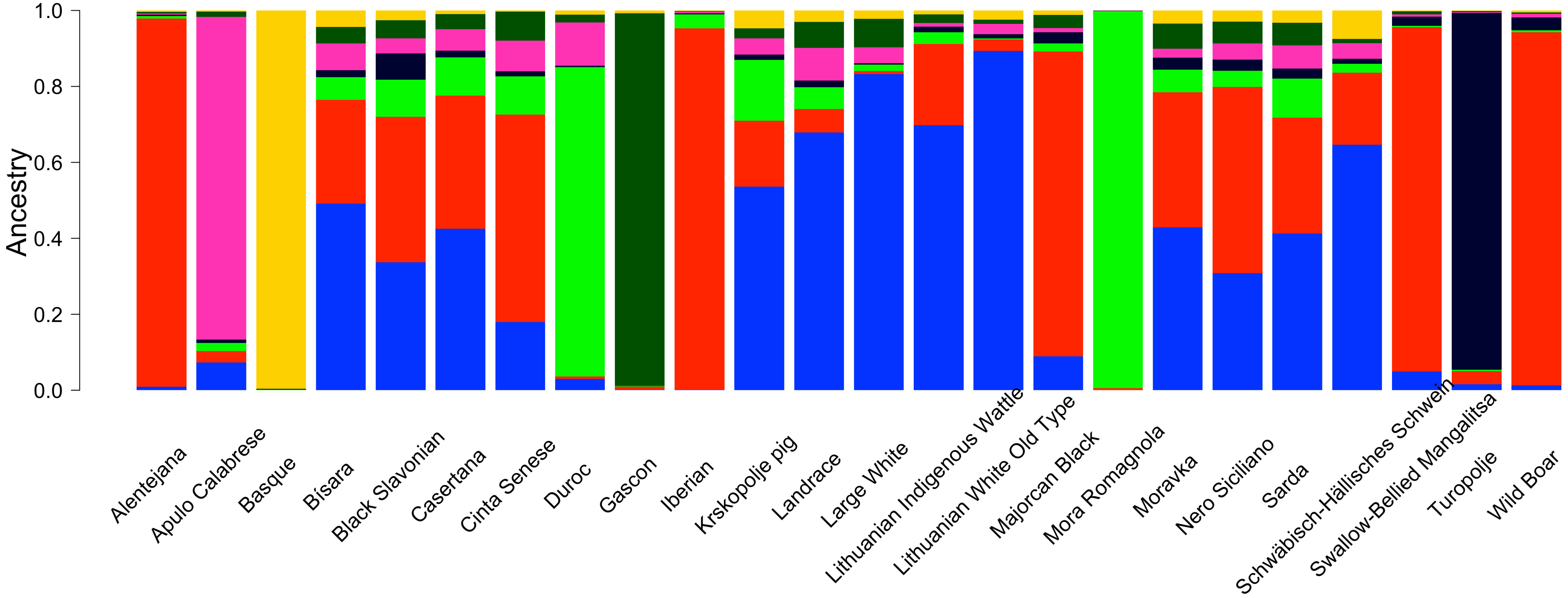

Supplementary Fig. S2. Change of average admixture ancestries per breed from K=8 to 24.

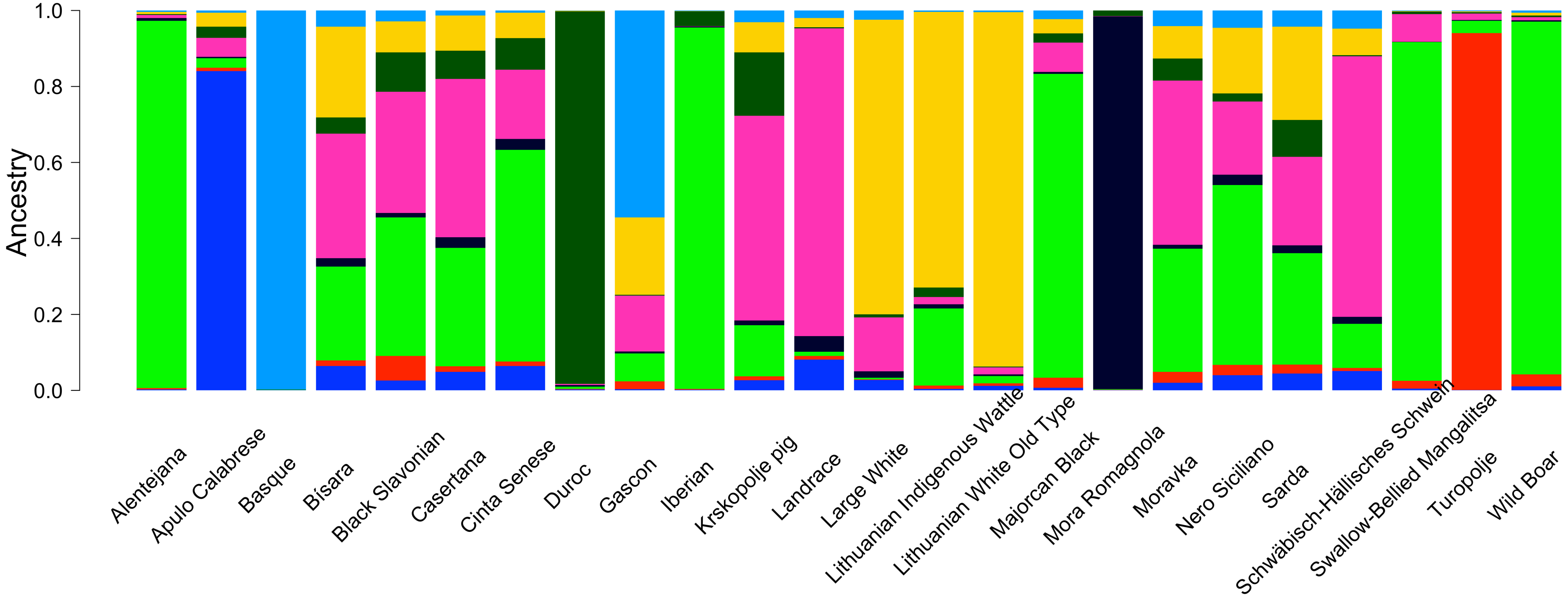

Supplementary Fig. S2. Change of average admixture ancestries per breed from K=9 to 24.

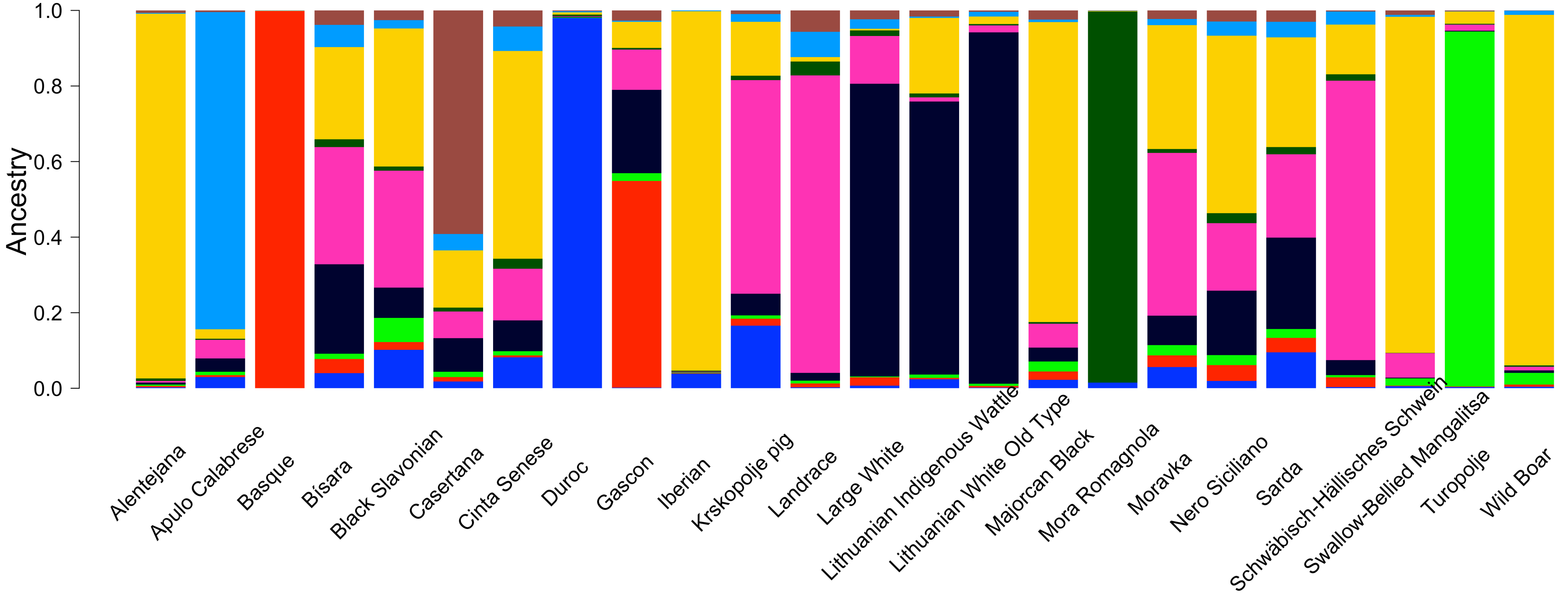

Supplementary Fig. S2. Change of average admixture ancestries per breed from K=10 to 24.

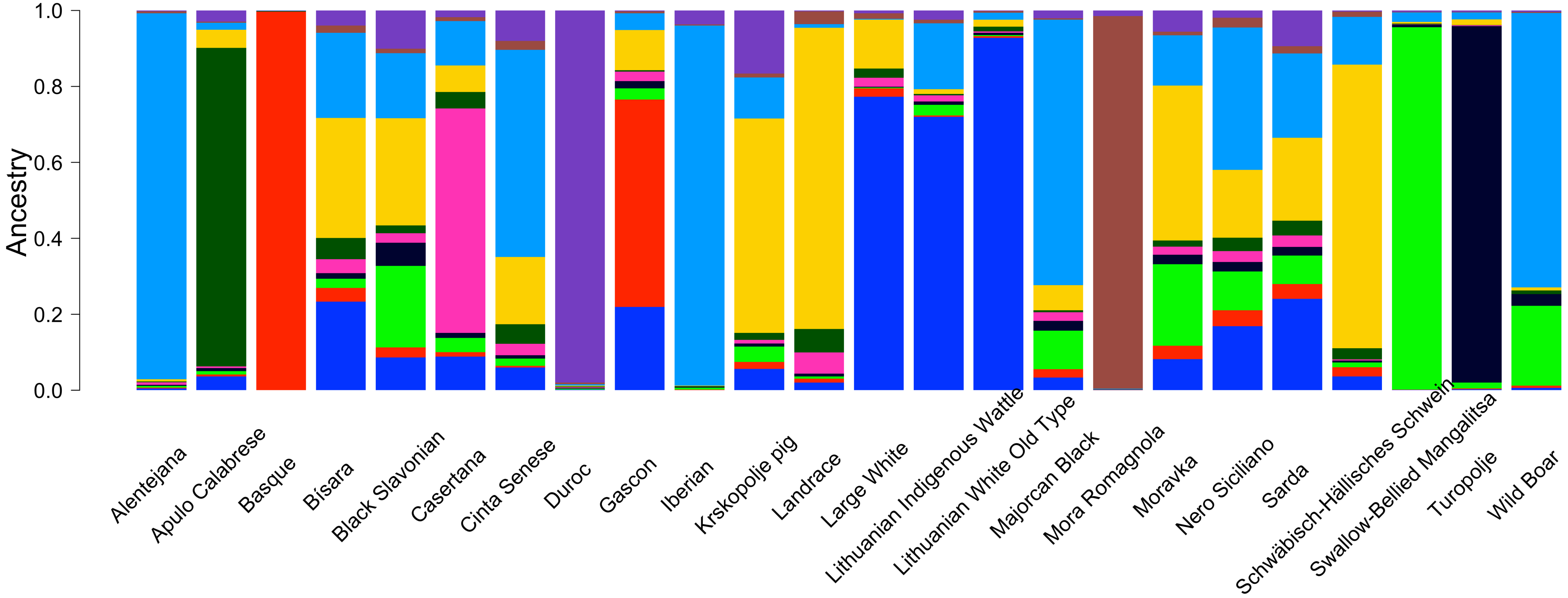

Supplementary Fig. S2. Change of average admixture ancestries per breed from K=11 to 24.

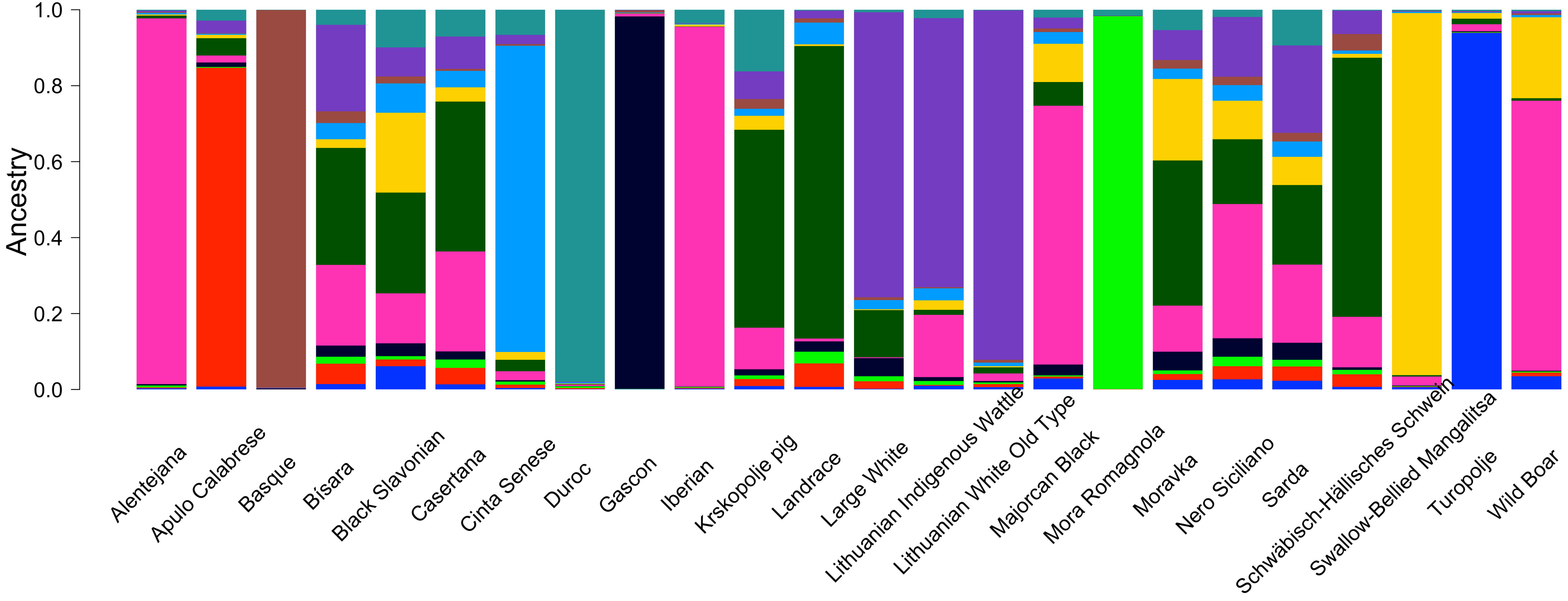

Supplementary Fig. S2. Change of average admixture ancestries per breed from K=12 to 24.

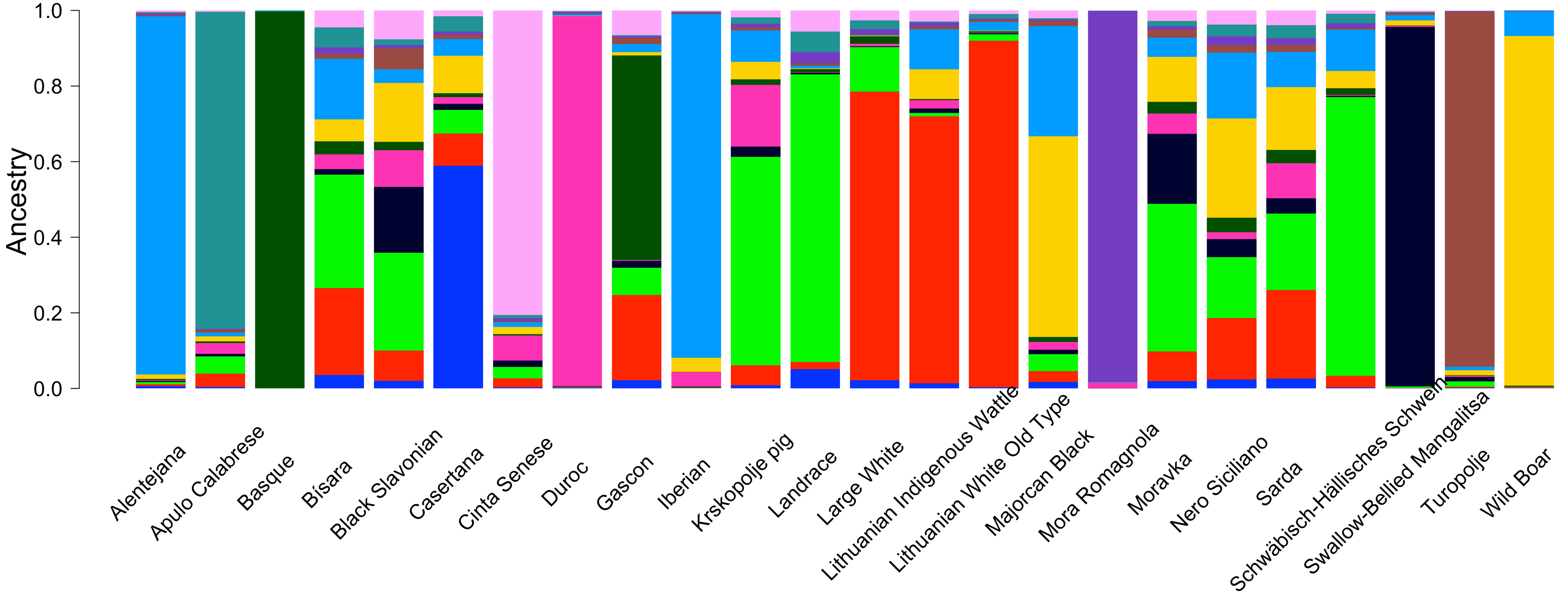

Supplementary Fig. S2. Change of average admixture ancestries per breed from K=13 to 24.

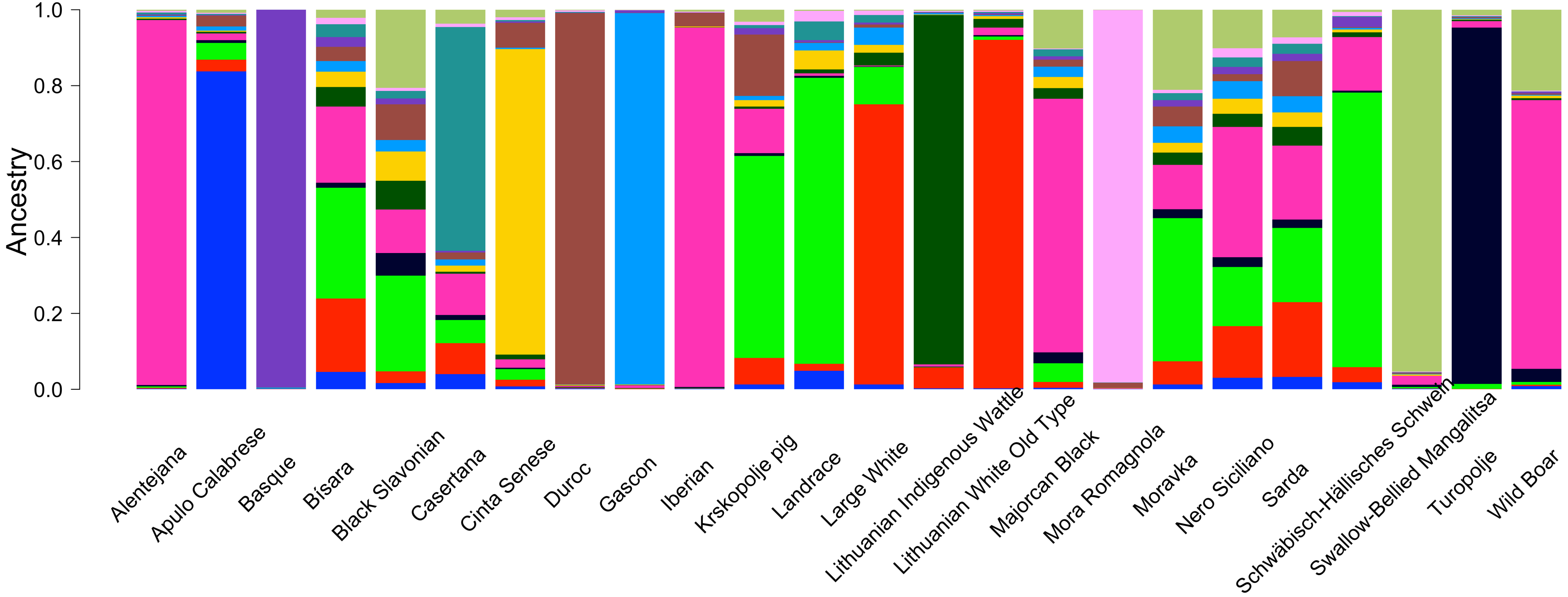

Supplementary Fig. S2. Change of average admixture ancestries per breed from K=14 to 24.

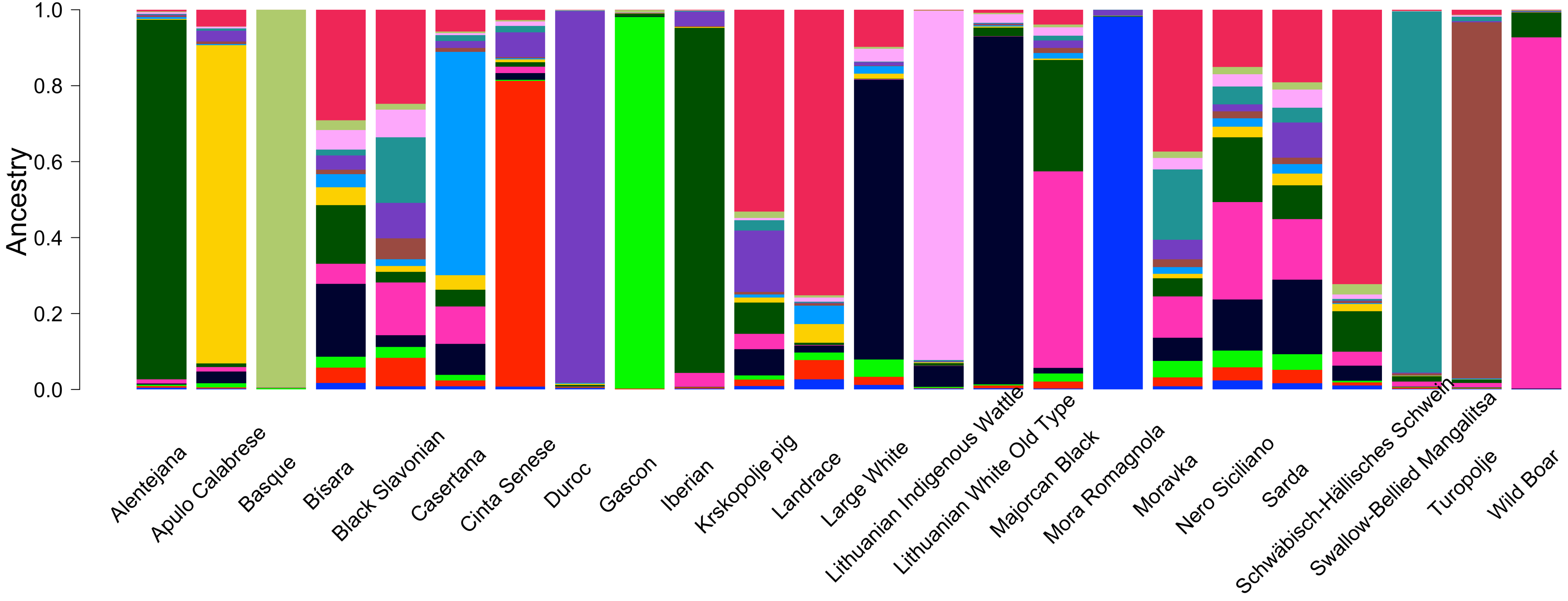

Supplementary Fig. S2. Change of average admixture ancestries per breed from K=15 to 24.

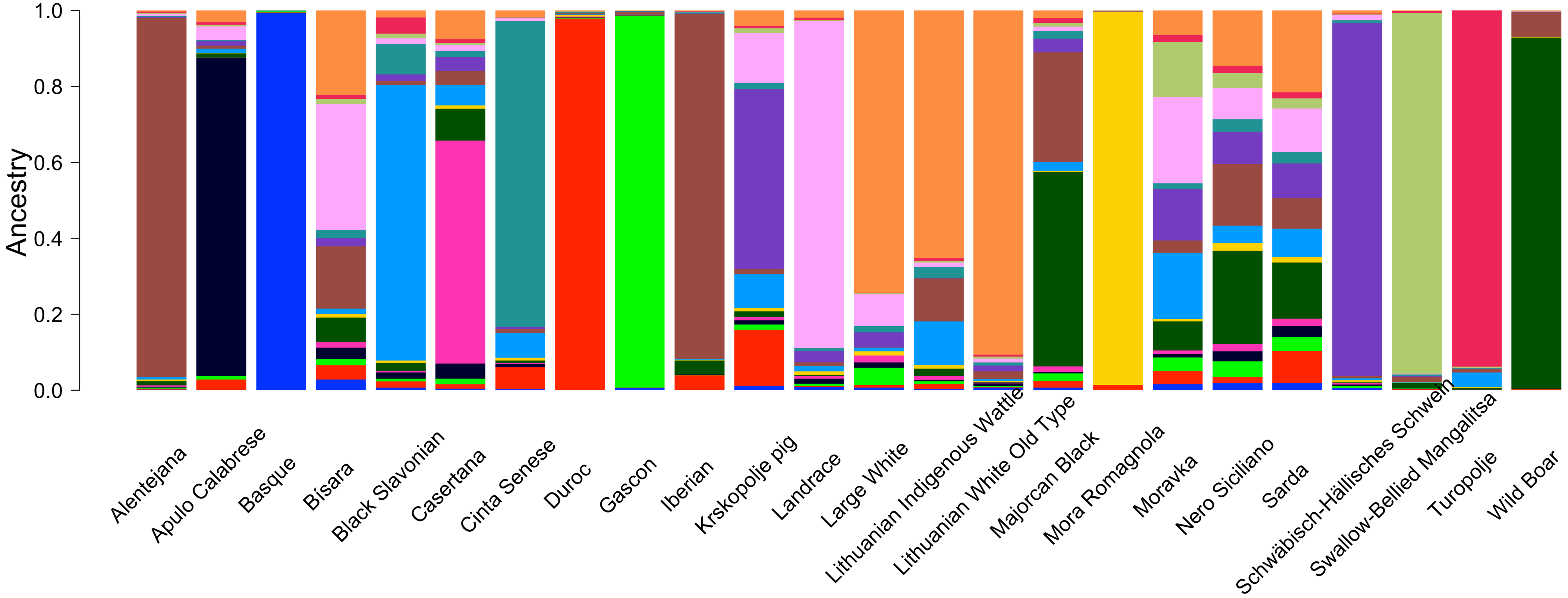

Supplementary Fig. S2. Change of average admixture ancestries per breed from K=16 to 24.

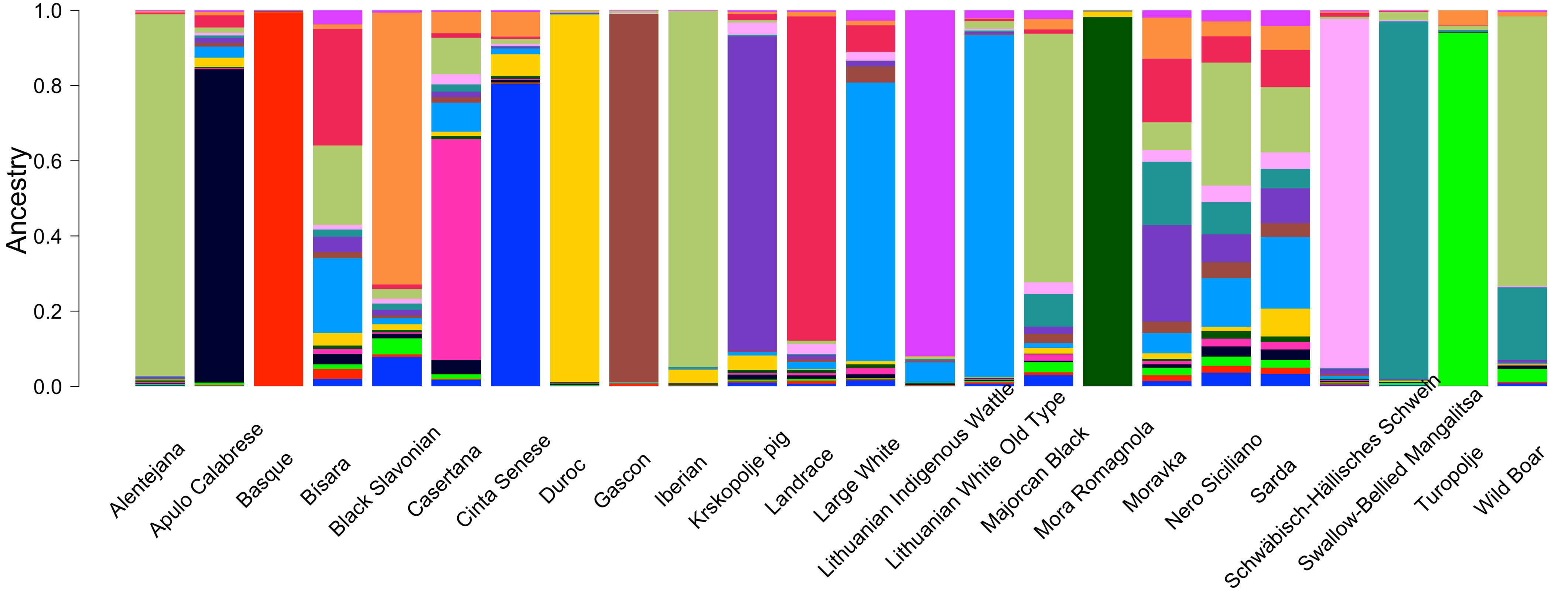

Supplementary Fig. S2. Change of average admixture ancestries per breed from K=17 to 24.

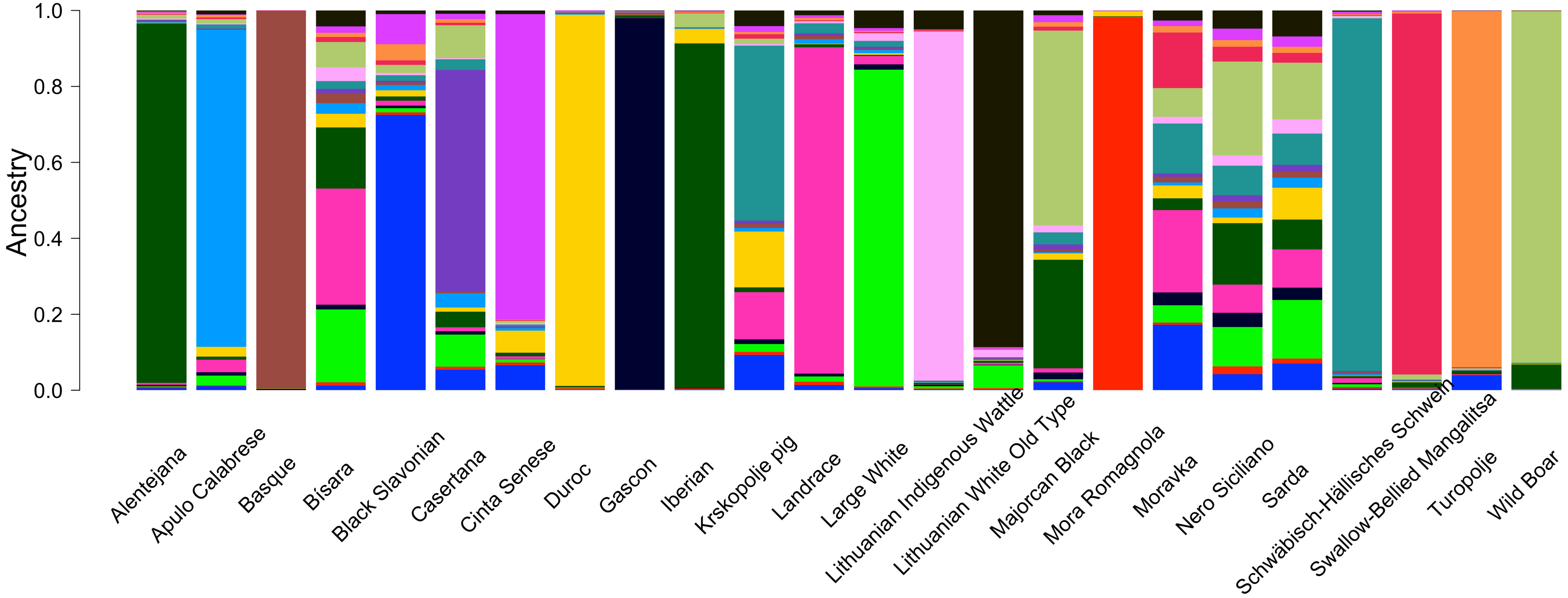

Supplementary Fig. S2. Change of average admixture ancestries per breed from K=18 to 24.

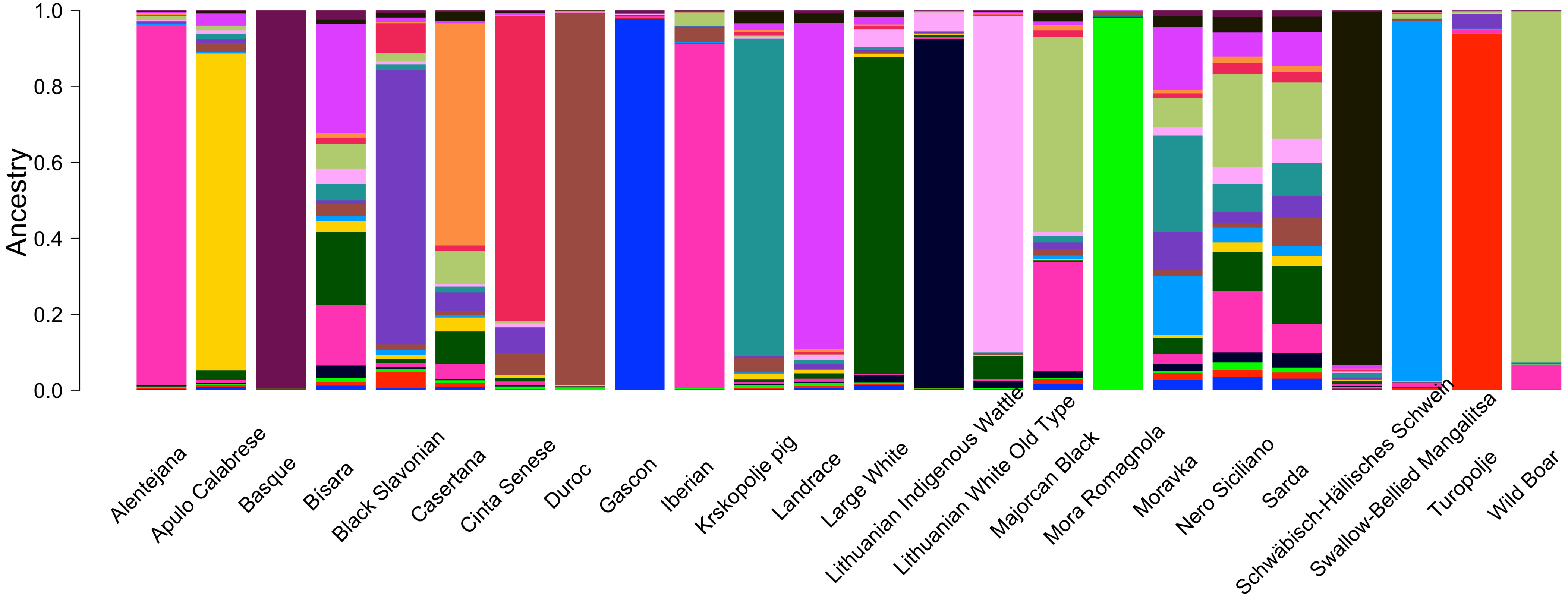

Supplementary Fig. S2. Change of average admixture ancestries per breed from K=19 to 24.

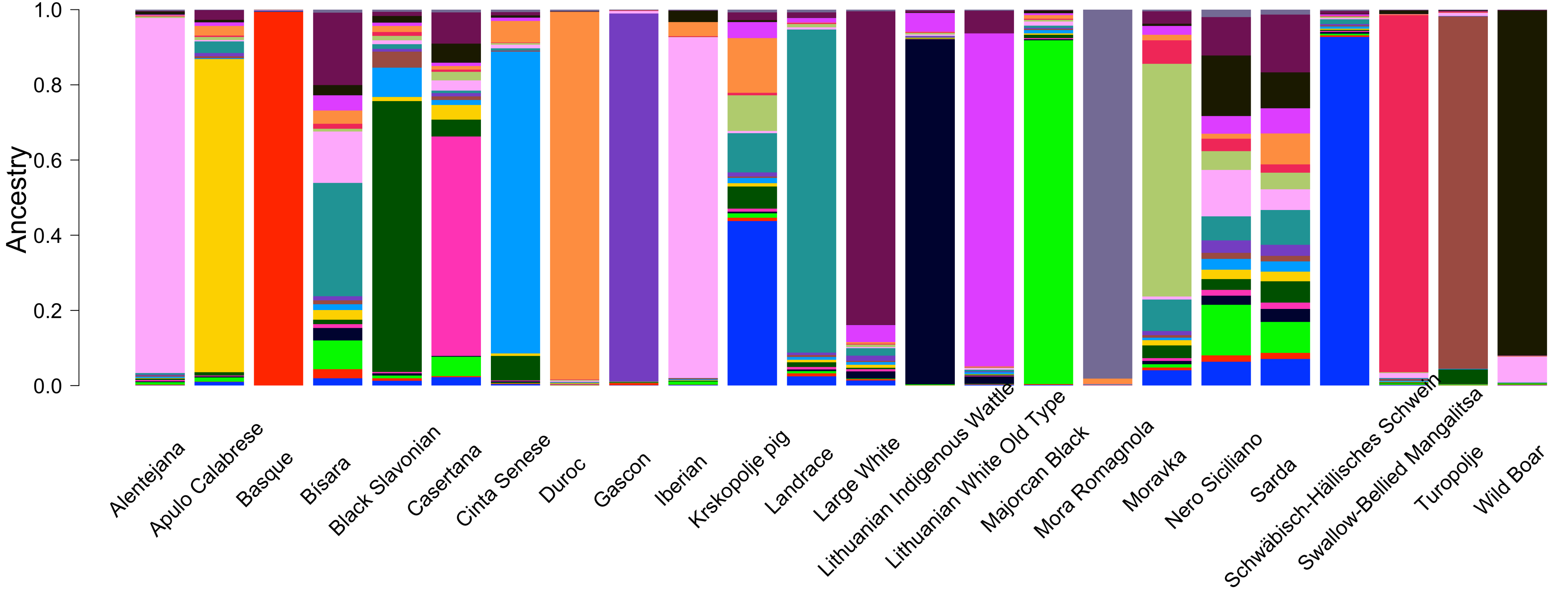

Supplementary Fig. S2. Change of average admixture ancestries per breed from K=20 to 24.

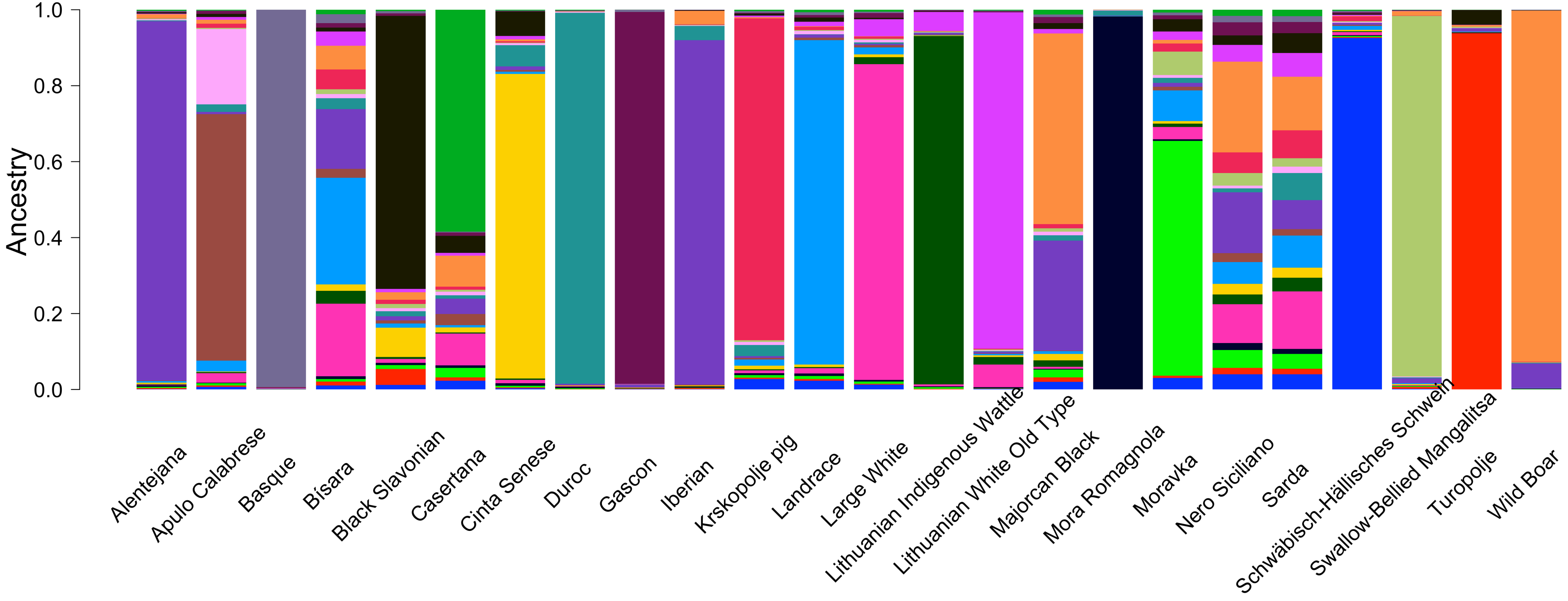

Supplementary Fig. S2. Change of average admixture ancestries per breed from K=21 to 24.

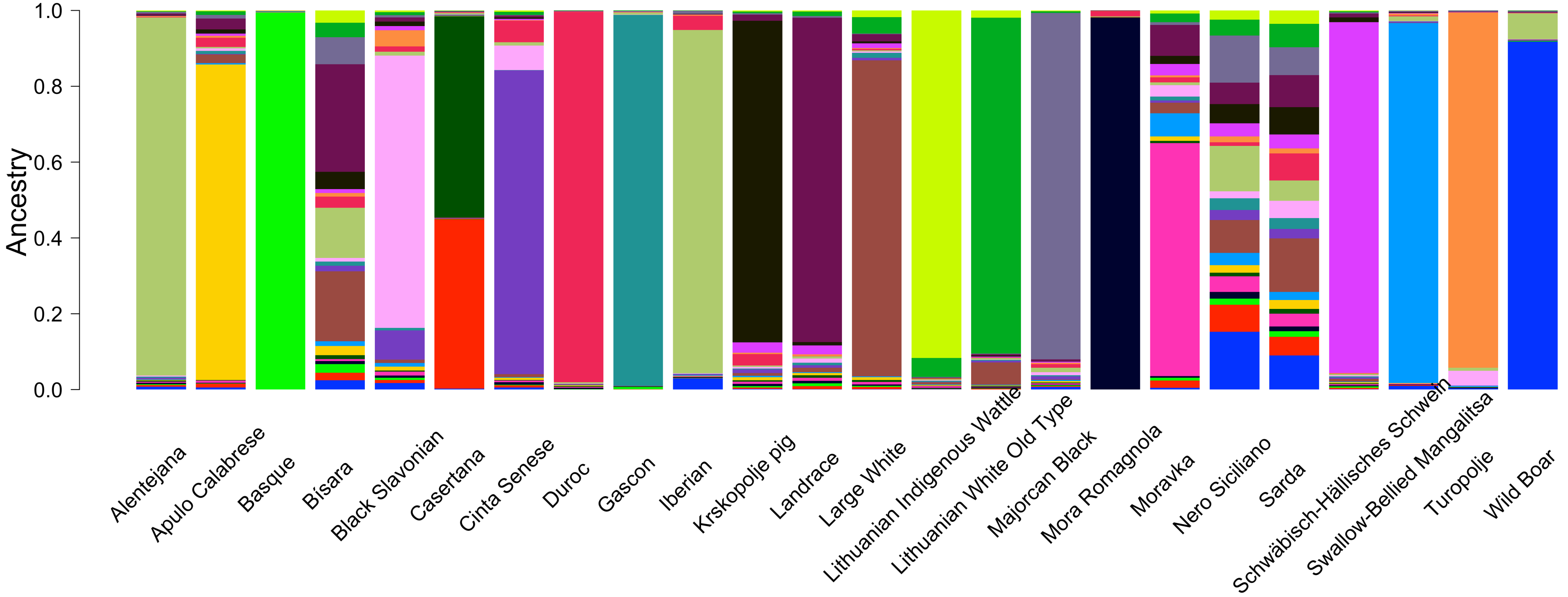

Supplementary Fig. S2. Change of average admixture ancestries per breed from K=22 to 24.

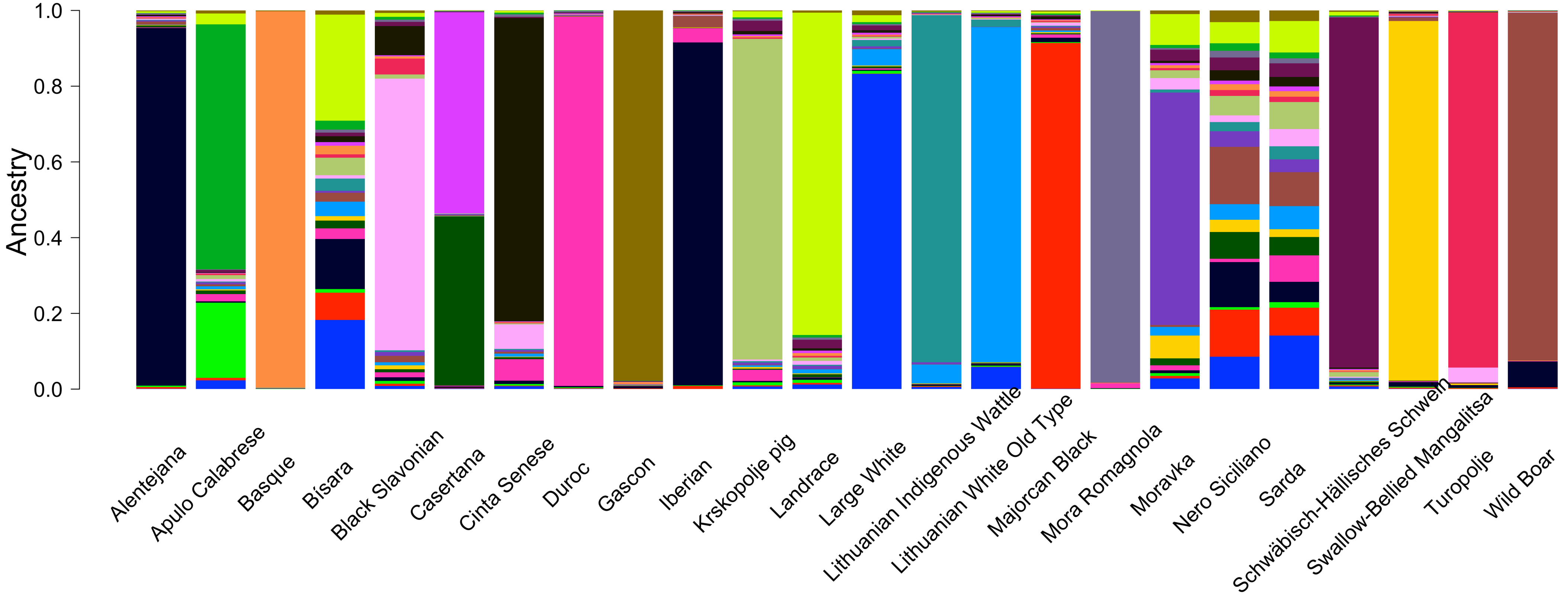

Supplementary Fig. S2. Change of average admixture ancestries per breed from K=23 to 24.

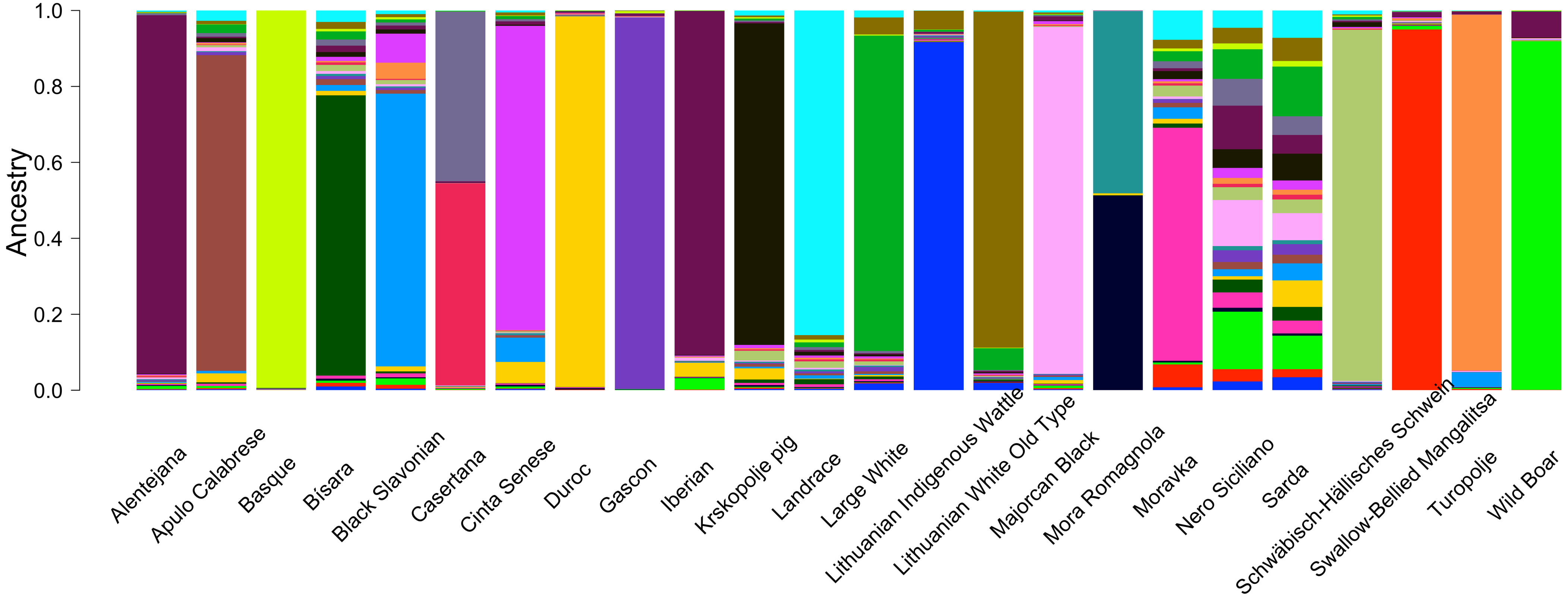

Supplementary Fig. S2. Change of average admixture ancestries per breed from K=24 to 24.

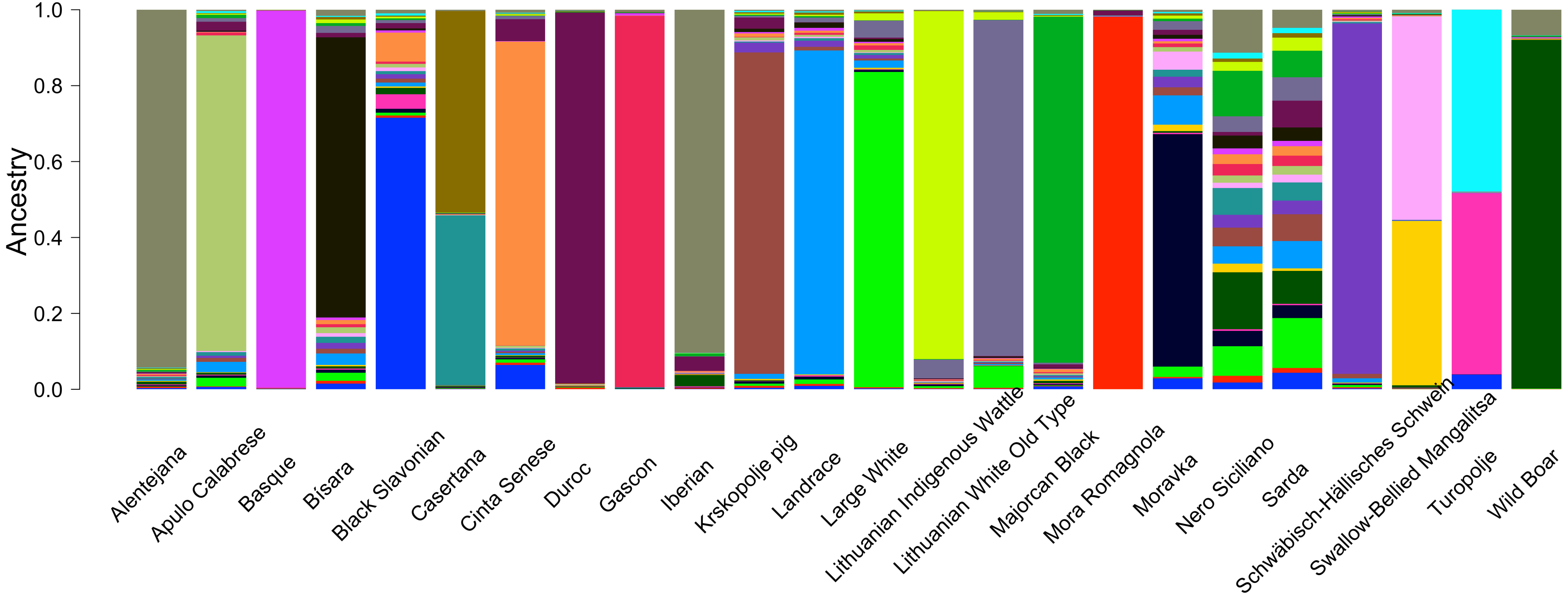

Supplement: Supplementary file 2 — Supplementary Information 2. [file 41598_2022_10698_MOESM2_ESM.pdf]
